# Computational Study of the Solid-State Incorporation of Sn(II) Acetate into Zeolite β

**DOI:** 10.1021/acs.jpcc.3c02679

**Published:** 2023-09-15

**Authors:** Owain T. Beynon, Alun Owens, Giulia Tarantino, Ceri Hammond, Andrew J. Logsdail

**Affiliations:** † Cardiff Catalysis Institute, Cardiff University, Park Place, Cardiff CF10 3AT, Wales, U.K.; ‡ Department of Chemical Engineering, Imperial College London, London SW7 2AZ, U.K.

## Abstract

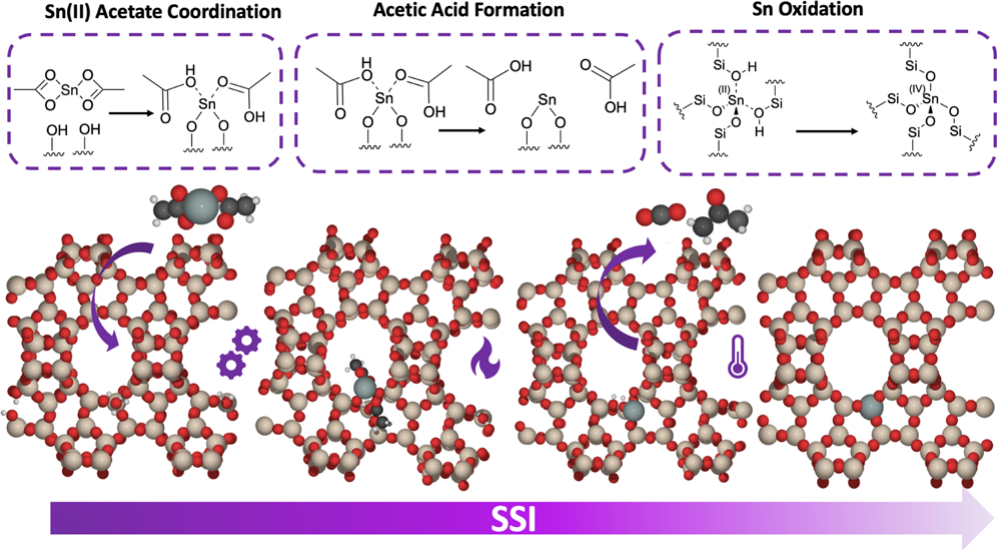

Sn-doped zeolites are potent Lewis acid catalysts for important reactions in the context of green and sustainable chemistry; however, their synthesis can have long reaction times and harsh chemical requirements, presenting an obstacle to scale-up and industrial application. To incorporate Sn into the β zeolite framework, solid-state incorporation (SSI) has recently been demonstrated as a fast and solvent-free synthetic method, with no impairment to the high activity and selectivity associated with Sn-β for its catalytic applications. Here, we report an *ab initio* computational study that combines periodic density functional theory with high-level embedded-cluster quantum/molecular mechanical (QM/MM) to elucidate the mechanistic steps in the synthetic process. Initially, once the Sn(II) acetate precursor coordinates to the β framework, acetic acid forms *via* a facile hydrogen transfer from the β framework onto the monodentate acetate ligand, with low kinetic barriers for subsequent dissociation of the ligand from the framework-bound Sn. Ketonization of the dissociated acetic acid can occur over the Lewis acidic Sn(II) site to produce CO_2_ and acetone with a low kinetic barrier (1.03 eV) compared to a gas-phase process (3.84 eV), helping to explain product distributions in good accordance with experimental analysis. Furthermore, we consider the oxidation of the Sn(II) species to form the Sn(IV) active site in the material by O_2_- and H_2_O-mediated mechanisms. The kinetic barrier for oxidation *via* H_2_ release is 3.26 eV, while the H_2_O-mediated dehydrogenation process has a minimum barrier of 1.38 eV, which indicates the possible role of residual H_2_O in the experimental observations of SSI synthesis. However, we find that dehydrogenation is facilitated more significantly by the presence of dioxygen (O_2_), introduced in the compressed air gas feed, *via* a two-step process oxidation process that forms H_2_O_2_ as an intermediate and has greatly reduced kinetic barriers of 0.25 and 0.26 eV. The results provide insight into how Sn insertion into β occurs during SSI and demonstrate the possible mechanism of top-down synthetic procedures for metal insertion into zeolites.

## 1 Introduction

Sustainable energy production and chemical manufacture are challenges of global importance, as society looks to mitigate the environmental damage caused by the use of fossil-derived hydrocarbons. In this respect, significant effort is currently being directed toward improving the sustainability of chemical production. Particular targets of high importance include improving the atom efficiency of established chemical processes, replacing harmful reagents with greener alternatives, synthesizing new catalysts with improved activity and selectivity, and developing new routes to desirable chemical products starting from renewable biomass materials. In the latter context, zeolites containing dilute quantities of isolated Lewis acidic active sites (henceforth, Lewis acidic zeolites) have demonstrated significant potential.

The traditional efficacy of zeolites in the catalytic refinement of petrochemicals by isomerization, cracking, hydrocracking, and reforming arises from the Brønsted acidity of the framework,^1^ which occurs when the cation compensating for the negative charge of the AlO_4_^–^ tetrahedra (T) is a proton. In contrast, Lewis acidic zeolites owe their catalytic performances to the doping of the SiO_2_ framework with heteroatoms such as Sn^4+^ and Ti^4+^. Doping with these heteroatoms can create catalysts for a variety of sustainable chemical transformation reactions (*Vide Infra*).^2−4^ Brønsted acidity is defined as the ability of a species to donate a proton, and a Lewis acid is defined as the ability of a species to accept electron density from a Lewis base. Therefore, zeolites can behave independently as Brønsted or Lewis acids, and also as bifunctional Brønsted/Lewis acid catalysts, depending on the location and nature of the heteroatom(s), and this flexibility presents opportunities to design new catalysts for reactions important for green and sustainable chemistry.

Among emerging Lewis acidic zeolite materials, Sn-β has demonstrated significant potential as a catalyst for a variety of sustainable chemical reactions. Sn-β is a crystalline, medium pore size zeolite possessing the BEA topology, within which small quantities of Si^4+^ atoms have been substituted for Sn^4+^. Initially, Sn-β was demonstrated to be catalytically active for the Baeyer–Villiger oxidation (BVO) of cyclic ketones to lactones, which is a critical step in the production of a variety of industrial polymer products.^2,5−7^ Notably, the use of Sn-β allowed the traditionally employed peracid-based oxidants, such as *meta*-chloroperbenzoic acid, to be substituted by the green oxidant, H_2_O_2_.^5,6,8,9^ More recently, Sn-β has also been shown to allow the BVO of renewable ketones, providing a facile route to produce bio-based lactones, and hence access to various bio-based polymers.^10^ Consequently, the applicability of Sn-β to catalyze the conversion of other carbonyl compounds has been considered, with Sn-β developed as a catalyst for the Meerwein–Ponndorf–Verley (MPV) transfer hydrogenation of various carbonyl compounds, including those of relevance to biomass conversion.^3,11−13^

The ability of Sn-β to activate carbonyl compounds has also been leveraged to facilitate the catalytic upgrading of sugars, most notably glucose, which is the monomer building block of cellulosic biomass.^14−16^ For example, several studies have demonstrated the high activity and stability of Sn-β for the low-temperature (<130 °C) isomerization of glucose to fructose,^16,17^ which is an important reaction of relevance to the food industry and future biorefineries. At higher temperatures (≥150 °C), Sn-β has also been shown to be an effective catalyst for the retro aldol fragmentation of glucose. This complex cascade process yields α-hydroxy esters such as methyl vinyl glycolate (MVG) and methyl lactate (ML), which have attracted significant industrial interest as platform molecules for renewable polymers.^18,19^ In each of the above cases, Sn-β has been shown to be more active and/or selective than alternative Lewis acidic zeolites, including TS-1 and Ti-β.^20−22^

Though Sn-β has shown promise toward catalysis of reactions that are important to the production of sustainable chemicals, challenges with respect to catalyst synthesis continue to prevent widespread uptake. Traditionally, Sn-β has been synthesized *via* hydrothermal methods^17,23−26^ and isomorphous substitution of heteroatoms into the β framework has also been achieved in order to introduce Lewis acidity.^20,21,27−29^ Early attempts for Sn-β synthesis *via* hydrothermal procedures used alkaline media and OH^–^ as a mineralizing agent,^9^ which resulted in successful Sn incorporation. However, unwanted Brønsted acid sites remained, which indicated that the framework contained many defects. Subsequent attempts used F^–^ as a mineralizing agent and successfully incorporated Sn into the β framework with less defects.^30^ While ultimately successful, long synthesis timescales (up to 40 days for crystallization) are necessary with this approach,^30^ and utilization of HF as a mineralizing agent poses significant challenges for scalability.

**Scheme 1:**
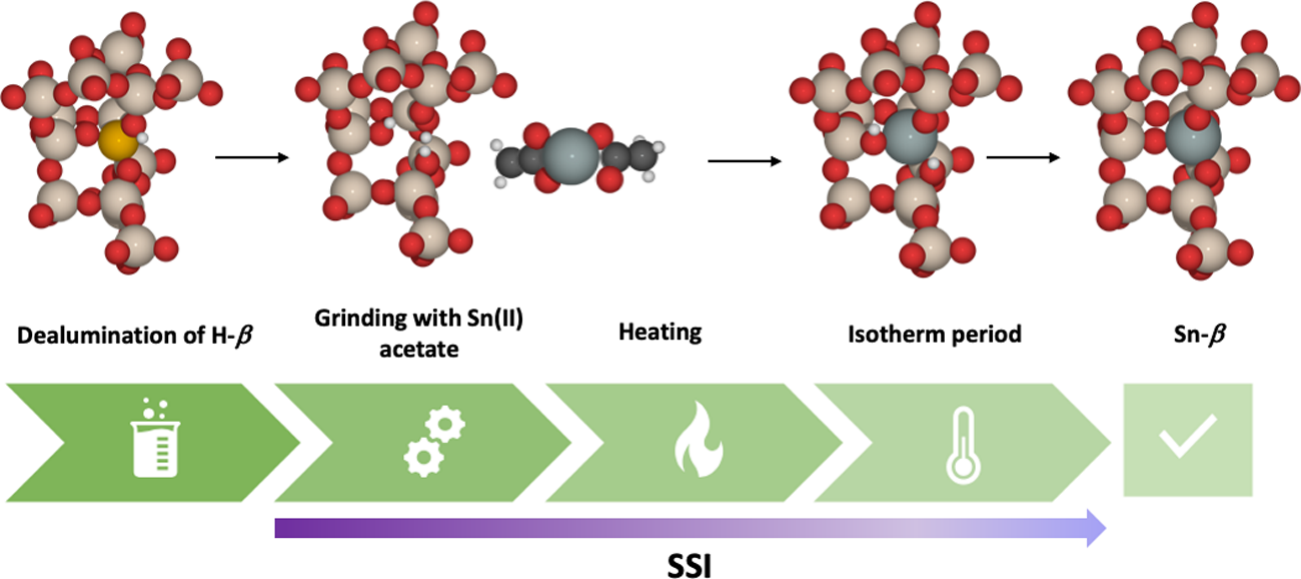
Schematic of Solid-State Incorporation (SSI) along with β Framework Species Formed The red, beige, white, black, orange, and gray atoms represent O, Si, H, C, Al, and Sn, respectively.

To overcome the highlighted limitations in the wet synthesis, solid-state incorporation (SSI) has been demonstrated as an alternative route to insert Sn into the β framework. SSI (Scheme 1) is a multistep process where active Sn-β catalysts are formed by the solid-state reaction between a Sn(II) precursor and a previously dealuminated β framework. Notably, dealumination of an aluminosilicate zeolite β with a strong acid (HNO_3_, 13 M) produces lattice vacancies in the form of hydroxyl nests, composed of four neighboring silanol groups (deAl-β). Subsequently, deAl-β undergoes physical grinding with Sn(II) acetate, and then the sample is heated to high temperatures (550 °C) in N_2_ (3 h) and air (3 h) before being allowed to cool, producing the final Sn^4+^ catalyst.^27,31−33^ In addition to demonstrating high performance as catalysts for the BVO and MPV reactions,^27,31−33^ Sn-β catalysts produced by SSI have shown excellent levels of activity, selectivity, and stability for glucose conversion processes,^34^ and recently established a new benchmark in performance for the retro aldol fragmentation of glucose.^35^ Furthermore, SSI can also achieve higher metal loadings than those feasible by traditional hydrothermal synthesis (≥2 wt %),^31−33,36^ and successful synthesis can be achieved in significantly shorter timescales (8 h). In combination, these properties make SSI an attractive route to producing Sn-β catalysts at large scale. Despite the efficacy of SSI as a method for Sn-β synthesis, a key challenge that remains is to maximize the fraction of Sn ions that effectively incorporate into the framework during preparation, resulting in the maximum number of active sites per mass of catalyst.

Despite the appeal to understand the SSI synthetic approach further, the incorporation mechanism of Sn into the zeolite lattice remains elusive, primarily due to the complexity of *in situ* studies, which has limited the quantity of mechanistic information available on this system.^33^ Elucidating the synthesis mechanism will aid the efficiency, and hence future scalability, of Sn-β synthesis, and consequently its catalyzed technologies, as well as providing insight relevant for SSI when applied to the synthesis of other Lewis acid zeolites. To support *in situ* mechanistic understanding, computational techniques have emerged as invaluable tools for the study of zeolites. Density functional theory (DFT) calculations have previously been employed to study heteroatom-doped β,^37−39^ with periodic boundary conditions (PBCs) and cluster models used to calculate the preferential site of heteroatom substitution in zeolites and measure Brønsted and Lewis acidity.^40−43^ Furthermore, hybrid quantum/molecular mechanical (QM/MM) studies have been employed for the study of zeolites and specifically β systems, as this technique addresses some of the shortcomings in periodic DFT and cluster approaches, such as high levels of metal substitution resulting from the periodic boundary conditions and the lack of consideration of long-range interactions, respectively.^44,45^ Studies have examined the stability of heteroatom incorporation into β and have reported the stability of the T2 site for Sn, which has been investigated using periodic DFT and QM/MM with agreement from experimental studies.^18,40,42,46,47^ Furthermore, Sn atoms substituted at the T2 site demonstrate higher Lewis acidity compared to other heteroatoms such as Ti and Zr.^37^ Although studies have noted the stability of Ti, the higher Lewis acidity of Sn supports the superiority of Sn-β for catalytic reactions.^40,42^ Moreover, computational investigations have also uncovered the higher hydrophobicity of Sn sites within β, which also aids in catalytic activity.^42^ The emphasis of these modeling efforts has been on framework properties of Sn-β, such as preferential T-site for Sn substitution, active site, and defect distribution,^40,48^ or catalytic reactivity,^43,49,50^ with little consideration of synthetic methods, especially top-down approaches.

Herein, we use computational approaches to investigate the mechanism by which Sn-β is synthesized *via* SSI. We build on previous work that combined *in situ* and DFT studies to elucidate the mechanism of Sn incorporation.^33^ In this work, we focus efforts on three key stages of SSI identified previously, namely, Sn(II) acetate incorporation, acetic acid ketonization, and Sn oxidation.

## 2 Computational Details

Periodic DFT calculations were performed with the “*Fritz Haber Institute ab initio molecular simulation*” (FHI-aims) software package, which is an all-electron, full-potential electronic structure code, and is suitable for periodic and nonperiodic systems.^51^ FHI-aims uses numeric atomic orbitals (NAOs) and calculations were performed using a “light” basis set of the 2010 release. The light basis set is equivalent to a double-numerical (DN) with polarization basis set and was chosen due to its converged accuracy and computational cost in benchmark testing (Figure S1A). The generalized gradient approximation of Perdew–Burke–Ernzerhof, reparametrized for solids (PBEsol), was used as the exchange-correlation functional,^52^ along with the method of Tkatchenko–Scheffler to account for dispersion corrections, which is a pair-wise additive approach to include van der Waals interactions within the system.^53^ PBEsol+TS displayed good comparative performance in our benchmark testing (SI, Table S1). Calculations were performed with a converged Monkhorst–Pack **k**-point^54^ sampling grid of 2 × 2 × 2 (Figure S1B). Self-consistent field (SCF) convergence was deemed complete when the change in electron density was below 10^–6^ e/a_0_^3^. Calculations were performed spin-restricted and using the zeroth order regular approximations (ZORA) for relativistic treatment.^55^

**Figure 1:**
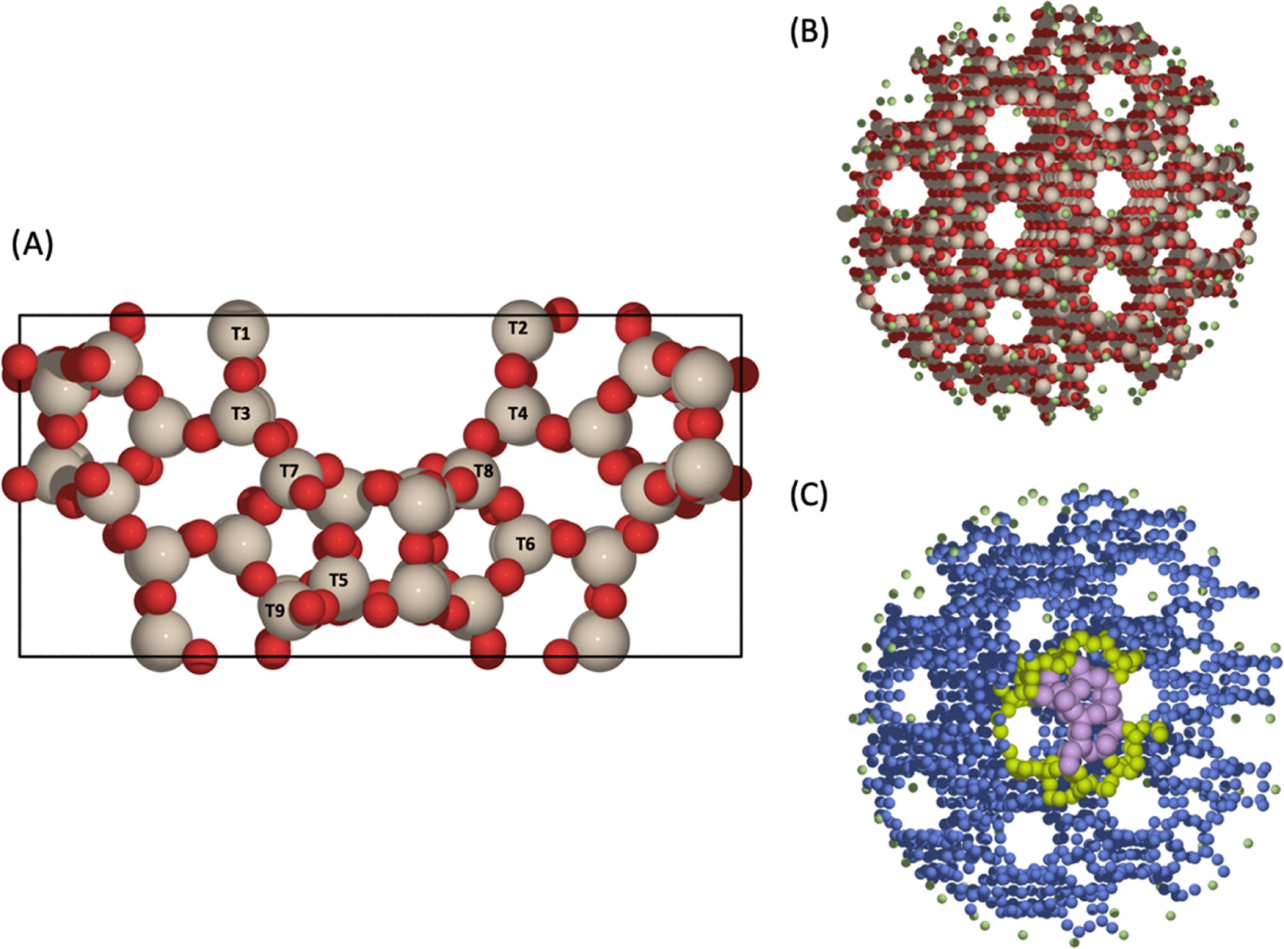
(A) Unit cell of zeolite β (BEA) along with the 9 distinct crystallographic tetrahedral (T-) sites labeled. Red and beige atoms represent O and Si, respectively. (B) QM/MM cluster model of Sn-β. Red, beige, white, and green atoms represent O, Si, H, and point charges, respectively. (C) Cross section of the cluster depicting regions of the cluster model treated by different theory. Purple, yellow, blue, and green spheres represent QM region, active MM region, frozen MM region, and point charges, respectively.

Structural models were managed using the Atomic Simulation Environment (ASE) Python library.^56^ An initial β unit cell (Figure 1A) was created from the structure first characterized by Newsam (192 atoms, *a*,*b* = 12.632 Å, *c* = 26.186 Å),^57^ and full geometry and unit cell optimizations were performed using the Broyden–Fletcher–Goldfarb–Shanno (BFGS) algorithm^58−61^ with convergence reached when the forces on all atoms are less than 0.01 eV Å^–1^. Subsequent geometry optimizations of atomic coordinates (*i.e.*, fixed cell) were performed on β-type structures along the SSI reaction scheme using the same algorithm and convergence criteria.

Transition state structures and minimum energy pathways were identified using a machine learning nudged elastic band (ML-NEB) method, implemented in the CatLearn Python library.^62^ The ML-NEB approach uses a Gaussian process regression to evaluate uncertainty estimates of all images in a reaction pathway and sets the uncertainty to be a criterion for convergence. Framework atoms not directly involved in the reaction pathway were frozen, as benchmarking showed the choice did not influence the reaction profile. Convergence was achieved when the average uncertainty in the interpolated pathway was below 0.05 eV and the force on individual atoms was below 0.05 eV Å^–1^, as deemed sufficiently accurate in benchmark testing (Figure S2).

To complement the periodic DFT, where stated a hybrid quantum mechanics/molecular mechanics (QM/MM) approach was used to calculate energetics and properties of β with higher-level hybrid-DFT and QM methods, using the Py-ChemShell package.^63^ Spherical embedded-cluster models of the Sn-β active site were created from the optimized unit cell of β (*i.e*., as used for periodic DFT studies) and centered around the symmetry distinct T2 position, for continuity with periodic modeling (Figure 1B).

The embedded-cluster models were treated by two levels of theory during calculations; a central QM region, which is the chemically active site of the model, and an encapsulating MM region, where long-range structural and electrostatic treatments are implemented to ensure correct bulk representation (Figure 1C). For the purposes of geometry optimizations, the MM region was subdivided into two regions: inner and outer MM regions, where atoms are free to move or constrained during optimizations, respectively. During calculations, the central QM region was defined as being up to the fifth nearest neighbors from the central atom (Figure S3), and the inner and outer MM regions extended to a radius of 10.5 Å (20 a_0_) and 26.5 Å (50 a_0_) from the central atom, respectively, where the latter is the radius of the entire cluster. These settings are aligned with prior QM/MM work on zeolite systems.^44,64,65^ The total number of atoms in the Sn-β cluster models was 3829, with 56 QM atoms, and 225 inner MM atoms (Figure 1B). All atoms in the QM region and the inner MM region were unconstrained during geometry optimization.

During QM/MM calculations, terminal oxygen atoms at the edge of the QM region were saturated with hydrogen atoms, where these “link” atoms ensure correct valency for the terminal atoms; bond-dipole corrections are also added at the boundary to ensure an accurate electrostatic-embedding potential.^66^ Models for Sn insertion reactions were “transposed” from periodic models into the Sn-β cluster model, *i.e.*, the starting structures were identical in both periodic and embedded-cluster models. For the transposition, all atoms within the embedded-cluster QM region were given the exact configuration of the periodic model, ensuring consistent energies with that of the periodic bulk systems (Figure S4).

To verify the accuracy of our QM/MM configuration, the energy of the QM region was calculated initially using PBEsol with the Tkatchenko–Scheffler dispersion correction (PBEsol+TS), *i.e.*, matching the periodic DFT calculations. Results were proven commensurate to 0.10 eV (Figure S4). Subsequently, the hybrid-DFT PBE0 functional^67^ was used with the TS correction (PBE0+TS), as well as second-order Møller–Plesset perturbation theory (MP2).^68^ All other QM calculation settings were identical to periodic models. The MM energy was calculated using GULP,^69^ employing the forcefield of Hill and Sauer,^70,71^ with the coordination-dependent charges in the original forcefield replaced with fixed point charges of 1.2 and −0.6 e for Si and O, respectively, as parameterized by Sherwood et al.^66^ Geometry optimizations were performed using DL-FIND^72^ as implemented within the Py-ChemShell package, using the limited memory BFGS (l-BFGS) algorithm. Structural convergence was assumed when the force on the atoms in the active QM and MM region were below 3 × 10^–4^ Ha/a_0_^3^ (0.01 eV Å^–1^)

To account for our atom-centered basis set, a basis set superposition error (BSSE) was calculated. The energies of Sn(II) acetate in the presence, *E*_A(A-Zeol)_, and absence, *E*_A(A)_, of the deAl-β basis functions were compared to the energy of deAl-β in the presence, *E*_Zeol(A-Zeol)_, and absence, *E*_Zeol(Zeol)_, of the Sn(II) acetate basis functions. The energy of the BSSE, *E*_(BSSE),_ was calculated *via*
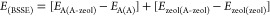
1With the “light” basis set, the BSSE for PBEsol+TS, PBE0+TS, and MP2 was calculated to be in the range of −0.03 to −0.06 eV (SI, Table S2), which is considered the error bar for each QM method going forward.

## 3 Results and Discussion

### 3.1 Sn(II) Coordination and Acetate Dissociation

**Figure 2:**
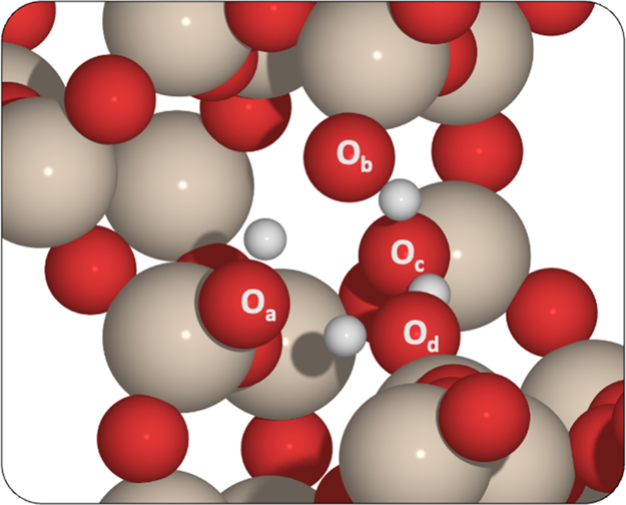
Silanol groups around the T2 position of BEA. Labels O_a_, O_b_, O_c_, and O_d_ represent the oxygen species in each of the hydroxyl moieties present. The red, beige, and white atoms represent O, Si, and H, respectively.

Early stages of SSI involve the grinding of Sn(II) acetate with deAl-β, which occurs prior to heat treatment. Initially, Sn(II) acetate must coordinate with the dealuminated framework in order to facilitate the subsequent transformation of Sn(II) acetate into the active Sn(IV)-β catalysts. Experimental studies report the formation of a silanol nest after dealumination with HNO_3_,^27,33,73^ where there are four silanol moieties with which the Sn(II) acetate can interact (Figure 2). The silanol nests occur on the distinct crystallographic T-sites previously occupied by Al; the nature and stability of these silanol nests in different crystallographic locations, along with doped heteroatoms within the framework, is widely discussed within the literature.^25,37,74,75^ Previous modeling efforts have focused on Sn-β with Sn incorporated on the T2 site,^37,39,40,42,46,47^ which has been reported as the most energetically favorable site for Sn substitution and with highest Lewis acidity. Furthermore, studies have reported the stability of T2 for Al occupation,^47^ and since the hydroxyl nests of the dealuminated framework are dependent on the sites previously occupied by Al, this strengthens the argument for modeling at the T2 site, which we pursue here. In addition, previous studies have reported that lower Sn loadings (<5 wt %) directly lead to active site formation, and although higher Sn loading achieves isomorphous substitution, this is accompanied by the co-formation of extra-framework Sn species that are not catalytically active.^33^ Therefore, in this work, we model one Sn atom per unit cell with a Sn to Si ratio of 1:63, consistent with experimental findings.

**Figure 3:**
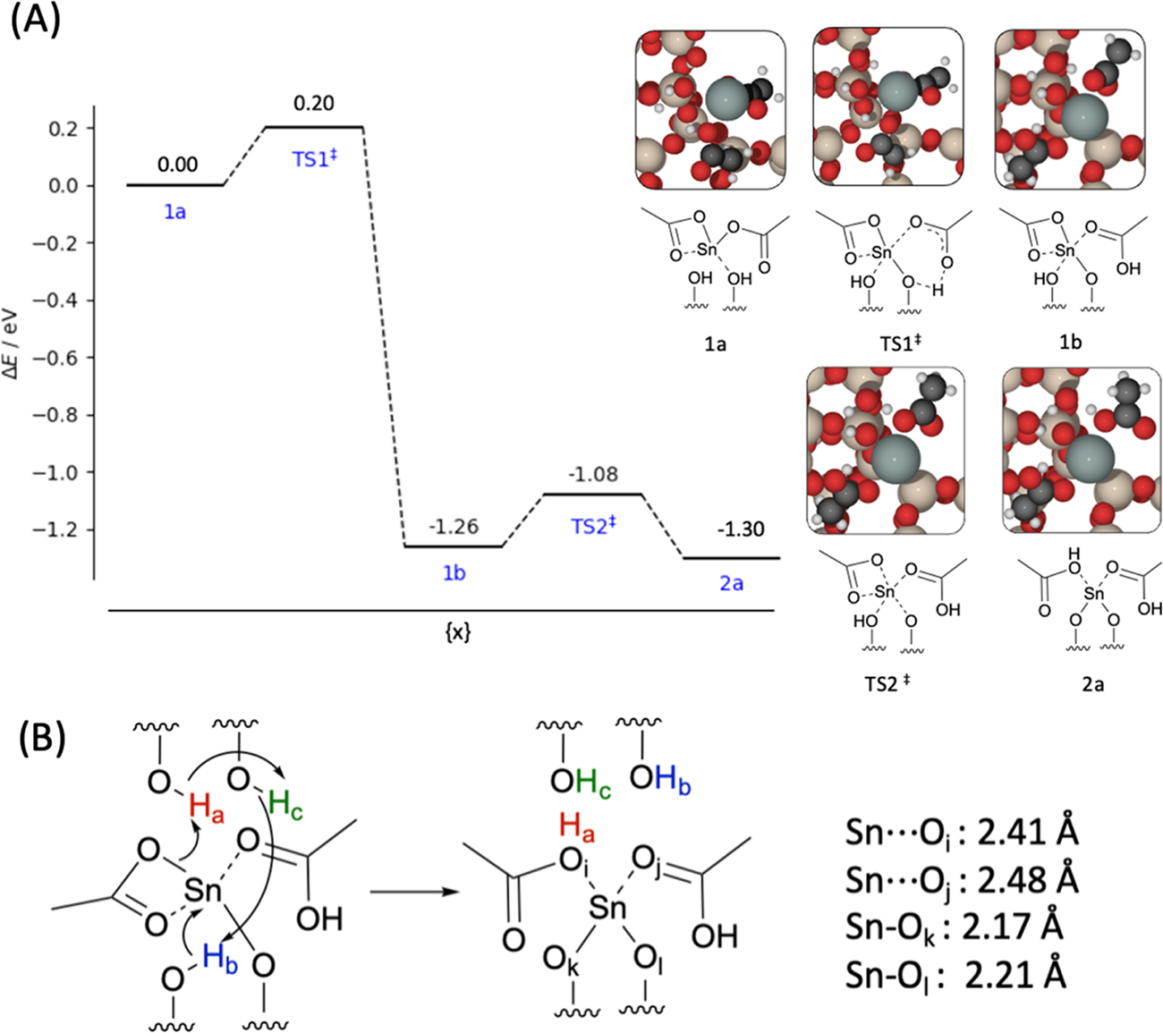
(A) Relative energy (Δ*E*) profile for the transformation of acetate ligand from bidentate (Structure 1) to monodentate (Structure 2) *via* TS1, with acetic acid formed *via* the transfer of H from a silanol of the framework onto the acetate ligand. Energies are calculated using periodic DFT. Insets show the structures at each respective geometry. (B) Scheme showing the concerted movement of H during the reaction, resulting in rearrangement of the remaining silanol moieties and H transfer from the framework onto the acetate ligand to form Sn-bound acetic acid, where the resultant Sn–O*_x_* distances are also noted. Red, beige, white, black, and gray atoms represent O, Si, H, C, and Sn, respectively.

**Figure 4:**
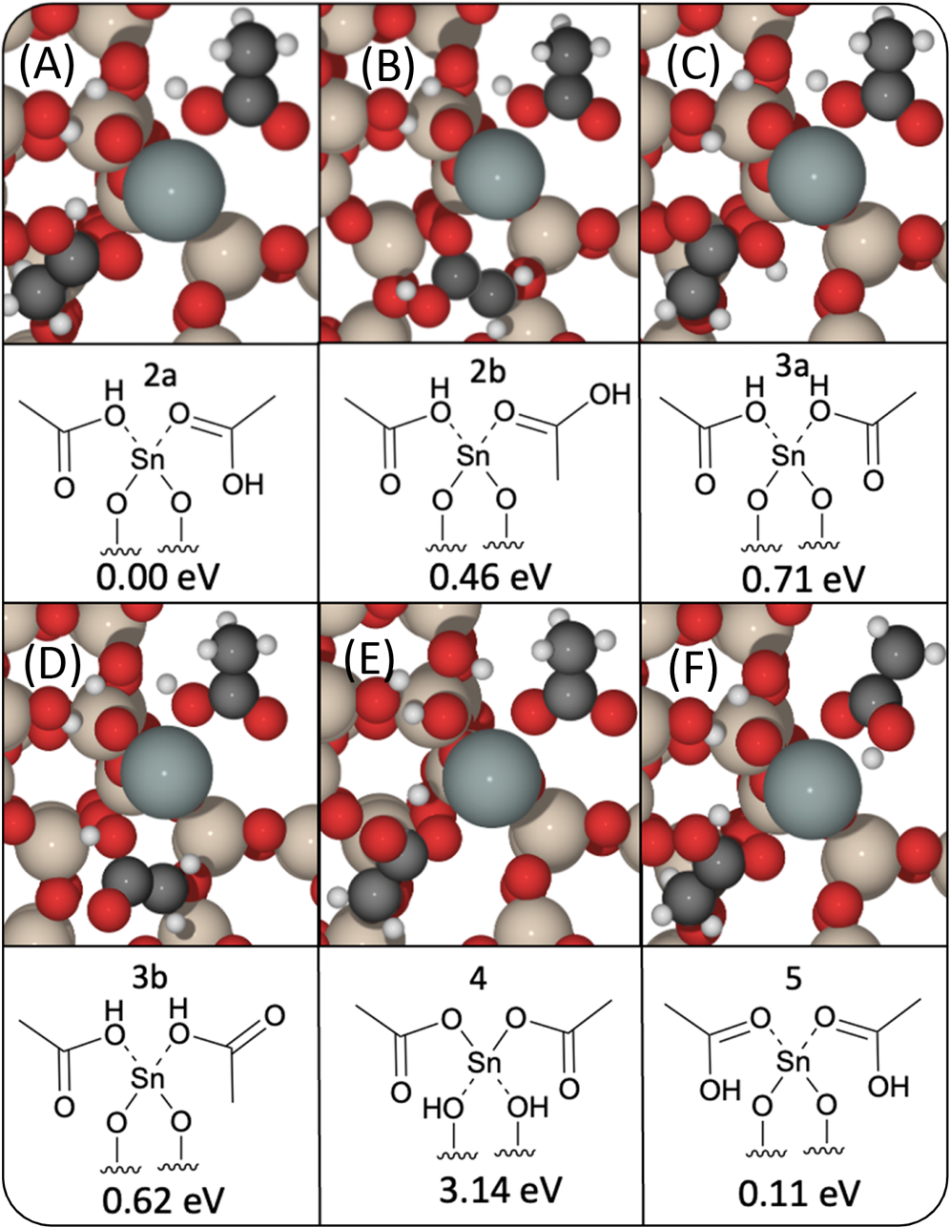
Permutations for bound acetic acid/acetate ligands, with structure number provided and stability (Δ*E*) relative to the lowest energy configuration. Energies are calculated using periodic DFT. In all cases, bonding to the framework occurs *via* the silanol nest; additional interactions are described for the labeled figures herein: (A) Bound Sn(II)(CH_3_COOH)_2_ with additional framework interaction *via* a [Sn(II)–(OH)–C] and a [Sn(II)–C–OH] connection. (B) Bound Sn(II)(CH_3_COOH)_2_ with additional framework interaction *via* a [Sn(II)–(OH)–C] and a [Sn(II)–C–OH] connection with a rotated acetic acid moiety around the Sn(II)–O–C bond. (C) Bound Sn(II)(CH_3_COOH)_2_ with additional framework interaction *via* a [Sn(II)–(OH)–C] connection. (D) Bound Sn(II)(CH_3_COOH)_2_ with additional framework interaction *via* a [Sn(II)–(OH)–C] connection with a rotated acetic acid moiety around the Sn(II)–(OH) bond. (E) Sn(II)(CH3COO)_2_ with no additional interactions. (F) Bound Sn(II)(CH_3_COOH)_2_ with additional framework interaction *via* a [Sn(II)–C–OH] connection. Red, beige, white, black, and gray atoms represent O, Si, H, C, and Sn, respectively.

**Figure 5:**
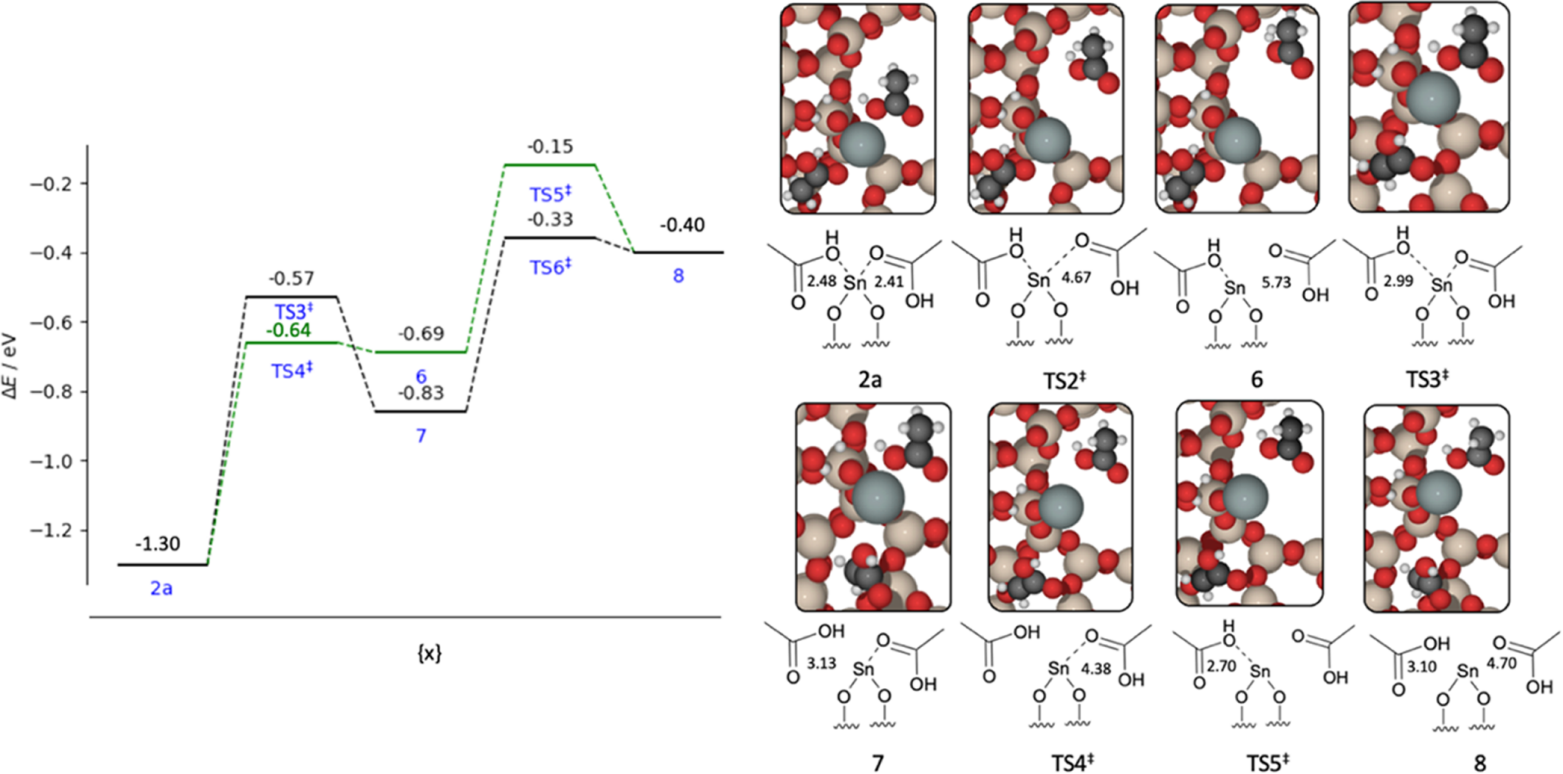
Graph of the reaction energies (Δ*E*) and kinetic barriers (*E*_act_) for the dissociation of the [Sn(II)–O–C(OH)CH_3_] (black) and [Sn(II)–(OH)C(O)CH_3_] (green) acetic acid ligands from structure 2a. Energies are calculated with periodic DFT. The *x*-axis represents the reaction coordinate, with numbers given in blue to identify the structural intermediates. Insets: visual representation of the structural intermediates, with structure numbers as labeled. Sn–O interatomic distances are given reported in angstrom (Å). Red, beige, white, black, and gray atoms represent O, Si, H, C, and Sn, respectively.

Experimental studies^33^ report a change in the coordination of Sn(II) acetate from bidentate to monodentate upon interaction with silanol groups of the deAl-β framework, while retaining a coordination number of 4, which we use as a foundation for our modeling. Additionally, our initial Sn(II) acetate model is a distorted trigonal bipyramidal due to a lone pair on the Sn atom, which is consistent with experimental studies (Table S3).^76^ Sn-oriented adsorption of Sn(II) acetate onto silanol oxygen O_a_ is favorable with an adsorption energy (*E*_ads_) of −0.40 eV in periodic DFT. Upon interaction with O_a_, one acetate ligand changes from bidentate to monodentate (Structure 1a, Figure 3A), which conserves the coordination number; from this configuration, the most accessible reaction is H transfer from a framework silanol to the monodentate acetate ligand (Structure 1b, Figure 3A). H transfer from the hydroxyl group of the framework stabilizes the monodentate configuration, which has a reaction energy (Δ*E*) of −1.26 eV and an activation energy barrier of 0.20 eV. The monodentate Sn(II) acetate is stabilized through coordination with 2 hydroxyl species in the framework, with a Sn(II)–O distance of 2.33 Å. From this configuration, further H transfer from a framework silanol to the second acetate ligand occurs, with a Δ*E* of −0.04 eV (*i.e.*, exothermic), and forms Sn(II)-bound acetic acid and a shorter direct Sn(II)–O framework bond of ∼2.20 Å (Structure 2a, Figure 3A). Evaluation of the kinetic barriers shows that the second H transfer occurs with the lowest barrier *via* a concerted mechanism, with an activation energy barrier of 0.18 eV (Figure 3A), and the hydrogen shuttling between hydroxyl species leads to a rearrangement of the silanol nests (Figure 3B). As the H transfer occurs, Sn(II) acetate converts fully from a bidentate to monodentate coordination. The H transfer from the zeolite framework to acetate ligand stabilizes a monodentate conformation; the acetic acid ligand is 3.14 eV more stable than monodentate acetate alone (Figures 4 and 5). The transfer of H from the framework to the acetate ligand, forming acetic acid, provides a pathway toward the incorporation of Sn(II) into the zeolite framework. Furthermore, opening up an acetate ligand, *i.e.*, transformation from bidentate to monodentate, in the β framework pore while uncoordinated to the silanol nest, and without H transfer, has a kinetic barrier of 0.80 eV (Figure S5), which is higher than the opening of the acetate ligand with the adsorbed Sn(II) acetate (0.18 eV). Therefore, the interaction with the framework greatly reduces the kinetic barrier for transformation between bidentate to monodentate Sn(II) acetate.

Studies have reported that the p*K*_a_ of acetic acid as 4.76,^77^ where reports in the literature have also noted the tunable p*K*_a_ of silanol groups in zeolites.^78−81^ The p*K*_a_ of a silanol group can be lowered to ∼4.5 when in close proximity to other silanol groups *i.e.*, a silanol nest.^79,80^ The literature therefore supports the simulation results in this work, where protonation of the acetate moieties by the silanol nest is observed. Furthermore, studies have also reported the stabilization of deprotonated silanols, which can lead to higher acidity.^82,83^ This is in contrast to orthosilicic acid, which has a higher p*K*_a_ of 9.5,^84^ suggesting that silanol groups in close proximity to each other, as with the silanol nest in deAl-β, can display high levels of acidity and plausibly protonate an acetate moiety to facilitate the formation of acetic acid as observed in simulations and in experimental studies of SSI.^33^

Furthermore, QM/MM results using MP2 indicate that the conversion from the bidentate Sn(II) acetate to structure 2a (Figure 3A) is notably exothermic with a Δ*E* of −3.21 eV (Figure S6 and Table S4), suggesting the formation of a monodentate species interacting with silanol groups of the frameworks is very favorable. The observation is most likely due to the formation of hydrogen bonding interactions between structure 2a and the β framework, which is absent in the case of the bidentate configuration. Moreover, energies calculated with MP2 are more exothermic than with periodic DFT, which is attributed to the higher accuracy of MP2 for describing these stabilizing interactions between the framework and the acetic acid ligands of structure 2a.

The framework-coordinated Sn(II) center can have monodentate acetate/acetic acid ligands with a variety of structures, as illustrated in Figure 4. The transfer of two H atoms from the silanol nest, as is necessary to stabilize the Sn(II) species on the framework, may result in hydrogenation of the carbonyl (C=O) bond or the single (C–O) bond, which creates a [Sn(II)–O–C–R] connection as with structure 5 (Figure 4F, Δ*E* = 0.11 eV) or a [Sn(II)–(OH)–C–R] connection, structure 3a (Figure 4C, Δ*E* = 0.71 eV), respectively; however, a mix between both [Sn(II)–O–C–R] and [Sn(II)–(OH)–C–R] connections, which we label structure 2a (Figure 4A, Δ*E* = 0.00 eV), is identified as the most stable arrangement with periodic DFT.

**Figure 6:**
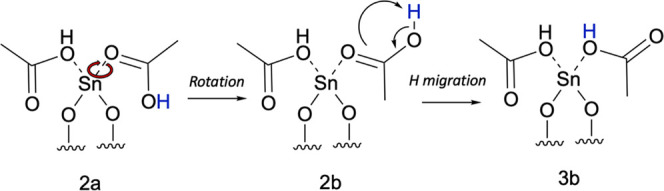
Scheme depicting the conversion of structure 2a to 2b *via* rotation of the Sn(II)–O bond, and conversion of 2b to 3b *via* H migration.

Once formed, the release of acetic acid from the Sn may readily occur. As reported in previous work,^33^ the separation of the acetic acid ligand bound through a [Sn(II)–(OH)–C–R] connection has an activation energy (*E*_act_) of 0.66 eV in periodic DFT, which is lower than for the [Sn(II)–O–C–R] alternative (0.73 eV), *i.e.*, the acetic acid ligand bound to the Sn through [Sn(II)–O–C–R] is more facile to remove (Figure 6). In this work, while considering the complete release of both acetic acid ligands, it is found that for structure 2a, the dissociation of the second acetic acid moiety is more facile, with a kinetic barrier of 0.56 and 0.54 eV for the [Sn(II)–(OH)–C–R] and [Sn(II)–O–C–R] connections, respectfully. Thus, the dissociation of the acetic acid moiety is overall easier and, given the relatively low kinetic barriers observed (<1 eV), it can be assumed that the formation of free acetic acid is facile under the reaction conditions of the heated stages in SSI (550 °C in N_2_ with a ramp rate of 10 °C/min) where acetic acid formation is detected,^33^ leading to strengthened interactions initially observed between Sn(II) and the β framework. Considering the release of acetic acid is an endothermic process with an overall Δ*E* of 0.90 eV (Figure 5), the reformation of an acetic acid moiety is also plausible. In that regard, given the small endothermic nature of the process, the heated reaction conditions, the potential entropic gains from the formation of molecular acetic acid, and that molecular acetic acid is a product observed during experiment, the formation of acetic acid can be identified as a key mechanistic step, and further kinetic modeling would be a valuable next step in the study.

**Figure 7:**
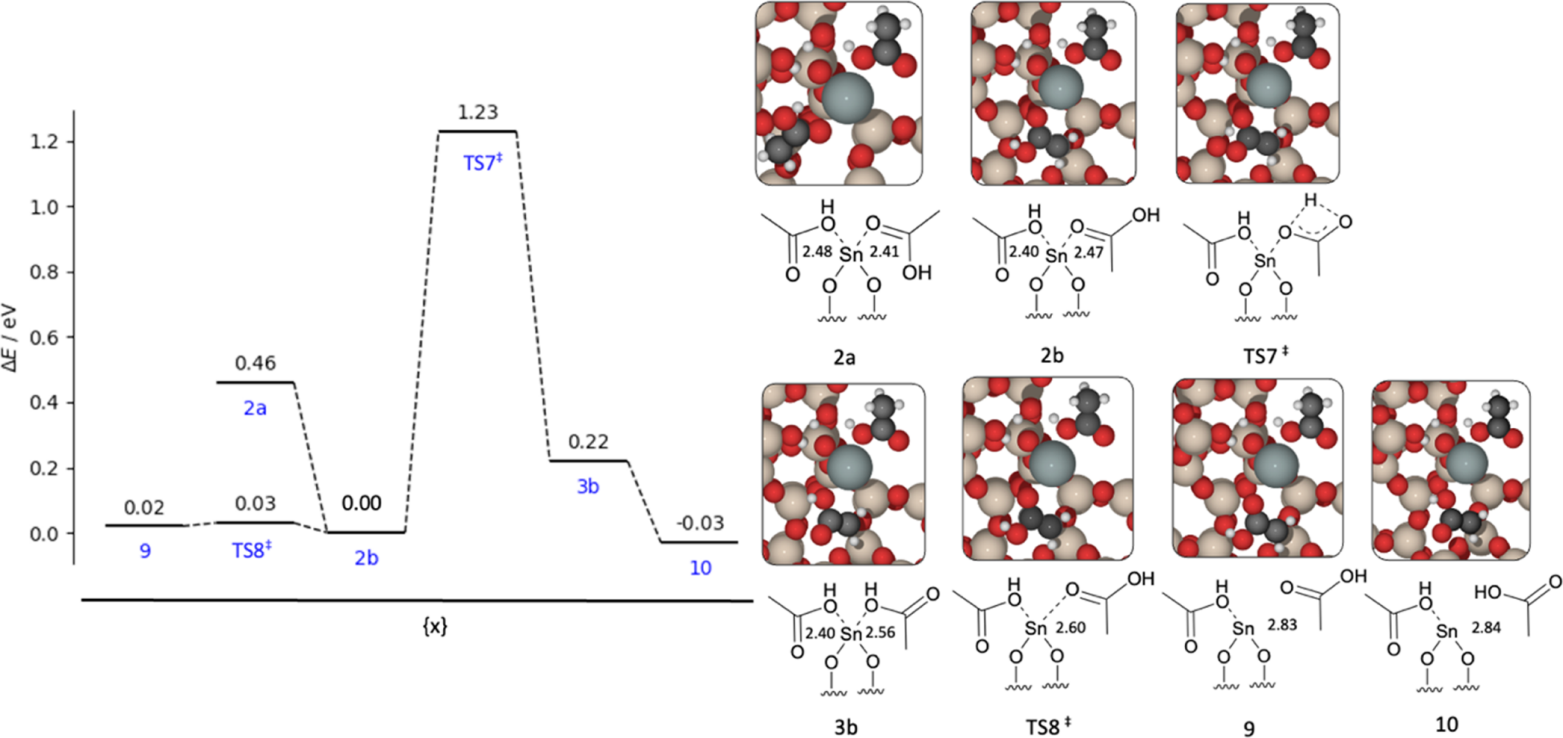
Graph of the reaction energies (Δ*E*) and kinetic barriers (*E*_act_) for conversion of structure 2a to 2b *via* rotation, and transformation of structure 2b to 3b *via* hydrogen migration, with subsequent dissociation of acetic acid moieties. Energies are calculated with periodic DFT. Insets: visual representation of the structural intermediates, with structure numbers as labeled. Sn–O interatomic distances are reported in angstrom (Å). Red, beige, white, black, and gray atoms represent O, Si, H, C, and Sn, respectively.

In addition to structure 2a, another conformer exists when bonded to the Sn(II) center, structure 2b (Figure 4B), where the relationship between both conformers can be described as similar to *cis* and *trans* substituents, as the conversion from 2a to 2b is through rotation around the Sn–O bond of the bound acetic acid ligand (Figure 6). Similarly, structure 3a has a conformer, 3b, which is also accessed through the rotation of the Sn–O bond (Figure 4D). The rotation of structure 2a to 2b has no kinetic barrier but is endothermic with a reaction energy of 0.46 eV (Figure 7) using periodic DFT, which suggests the facile interconversion between both conformers. QM/MM calculations with MP2 indicate that the conversion between 2a and 2b has a Δ*E* of −0.04 eV (Figure S6 and Table S4), which is slightly exothermic and contrasts with the results with periodic DFT. The calculated discrepancy could again be attributed to the ability of high-level methods such as MP2 to describe stabilizing interactions, owing to a more accurate treatment of electronic structure. Furthermore, the conversion between both conformers 2a and 3b is thought to be facile given the low energy nature of the process (<1 eV).

When investigating the role of acetic acid conformation on dissociation from the metal center, both the conformers 2b and 3b have lower kinetic barriers than 2a, being 0.00 and 0.03 eV with periodic DFT for dissociation from structures 3b and 2b, respectively. The lower energy barrier to form free acetic acid from the [Sn(II)–(OH)–C–R] connection in 3b is consistent with the observations for conformer 2a. Overall, as conformers 2b and 3b have lower kinetic barriers for acetic acid dissociation, the conversion from 2a to 2b may be a necessary step; however, entropic effects may also be a factor.

While considering the properties of the conformers, the interconversion of conformer 2b to 3b was considered *via* a H transfer mechanism. The process has a high kinetic barrier of 1.23 eV (Figure 7) with periodic DFT; the barrier can be substantially lowered to 0.29 eV if H_2_O is considered present, as H shuttling facilitates the reaction (Figure S7). However, given the high kinetic barrier for H transfer across the acetate ligand, and the assumed dehydrated nature of the framework, the conversion between 2b to 3b is deemed unlikely given the lack of heating in the grinding stages of SSI (which occurs at room temperature).

### 3.2 Acetic Acid Ketonization

**Figure 8:**
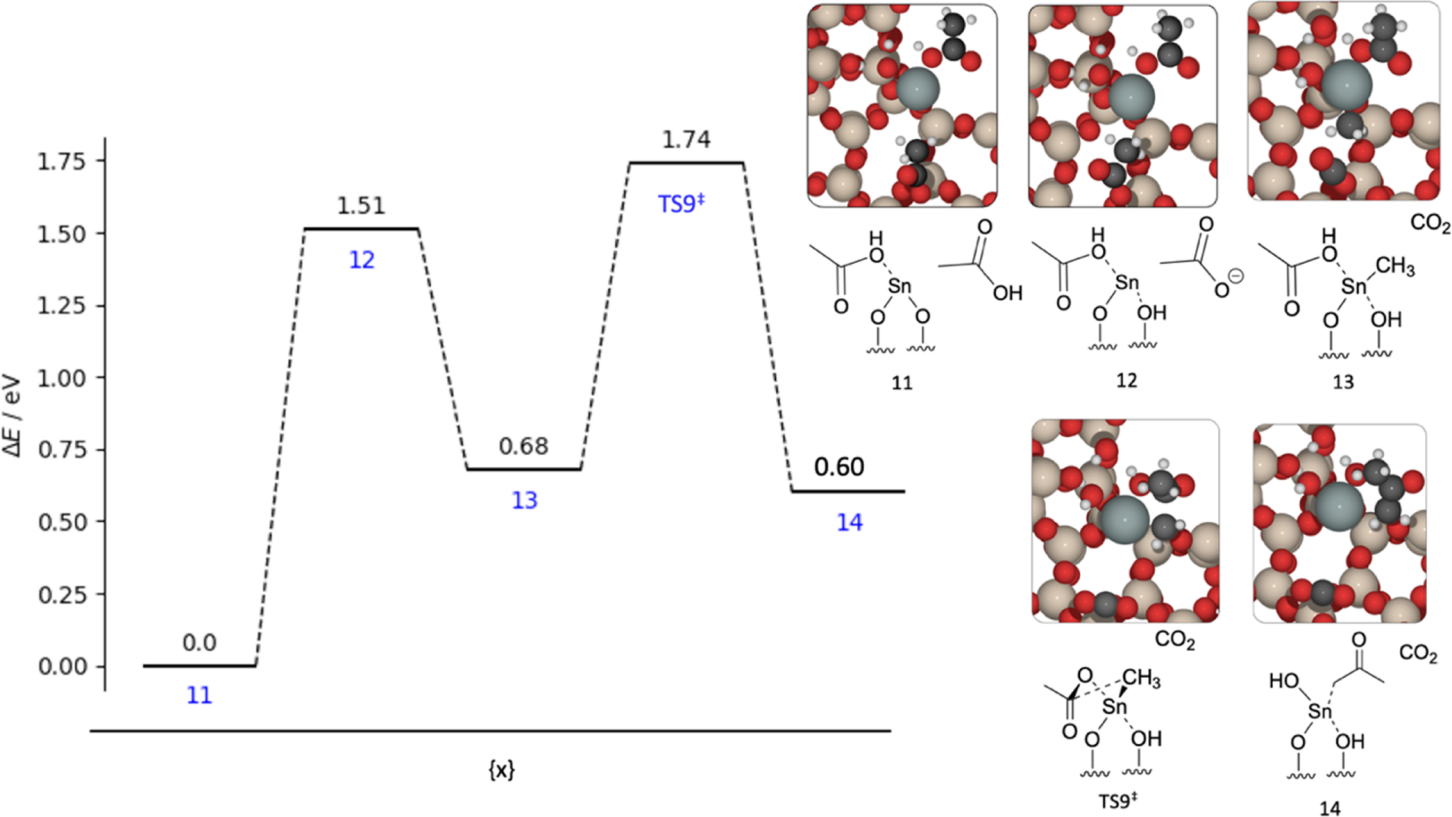
Graph of the reaction energies (Δ*E*) and kinetic barrier (*E*_act_) for ketonization of acetic acid to acetone and carbon dioxide across Lewis acid Sn(II) site. Energies are calculated with periodic DFT. Insets: visual representation of the structural intermediates, with structure numbers as labeled. Red, beige, white, black, and gray atoms represent O, Si, H, C, and Sn, respectively.

Acetic acid has been demonstrated as a facile product during Sn coordination to the β framework, but experiments report that both carbon dioxide and acetone are produced during the heating stages of SSI, which suggests that acetic acid may react further post-dissociation.^33^ Although the formation of acetic acid has been demonstrated as essential mechanistic steps for the incorporation of Sn into the β framework, the ketonization of acetic acid is not thought to play a direct role in Sn incorporation but provides information on the product distribution observed during SSI by TPD-MS. Experimental studies indicate that the mechanism for ketonization is dependent on the initial Sn(II) acetate loadings, where Sn loadings ≥5 wt % are proposed to present adjacent acetate moieties that could undergo ketonization. However, for lower Sn(II) acetate loadings studied in this work, the absence of adjacent Sn(II) acetate groups necessitates the consideration of other pathways for CO_2_ and acetone formation. Furthermore, the ketonization of acetic acid in zeolites, producing acetone and carbon dioxide, is well reported in the literature;^85,86^ however, the ketonization reactions typically occur over Al T-sites in Brønsted acid zeolites^87^ and these sites are not readily available in the SSI due to the dealuminated nature of the framework. In the case of synthesizing Sn-β, alternative pathways for ketonization must be considered, where dissociated acetic acid molecules could react in the zeolite pore or over the Lewis acidic Sn center (Figure 8).

The gas-phase ketonization of two acetic acid molecules to acetone, carbon dioxide and water, has a calculated energy barrier of 3.86 eV (Figure S8), which is too high to proceed under normal conditions and suggests that interaction with the framework is needed to mediate the reaction. Considering the earlier steps in the mechanism of SSI, where the formation of acetic acid from acetate *via* hydrogenation is facile, it is interesting to consider the reverse process where acetic acid could dehydrogenate to reform an acetate species, which is barrierless, and then interacts with the Lewis acid Sn(II) site (Figure 8). Subsequent scission of the acetate C–C bond occurs to produce carbon dioxide and a Sn-bound methyl group; the methyl group then reacts with a second acetic acid molecule, forming acetone over the Sn site, which dissociates leaving a Sn(II)–OH species. A maximum kinetic barrier of 1.06 eV (Figure 8) is calculated on this pathway when using periodic DFT. The Lewis acidity of the Sn site further supports the plausibility of this pathway, and the lower transition state energy relative to the gas-phase transformation indicates that the formation of acetone over the Sn is more feasible, especially considering the temperature conditions at this stage of the process (≥325 °C). However, for the formation of the initial acetate species, the endothermic nature of the process may restrict the thermodynamic accessibility of the product. Calculations indicate that interaction with Sn alleviates the barriers for ketonization, but our calculations are restricted to low concentrations of acetic acid molecules due to our initial decision to model low Sn(II) acetate loadings. In this regard, the further exploration of pathways toward the formation of CO_2_ and acetone is worth consideration, especially for higher loadings of Sn(II) acetate where alternative or even competing mechanics for ketonization may be present.

### 3.3 Sn Oxidation

Following Sn association with the zeolite framework, and subsequent ligand removal, the framework-inserted Sn(II) center (Structure 15) must undergo a change in oxidation state to form Sn(IV), the catalytically active state (Structure 16). The high-temperature oxidative conditions applied in the last stage of the SSI (550 °C in air flow) are proposed to result in framework dehydrogenation,^33^ where Sn–(OH)–Si connections are transformed to Sn–O–Si. As each hydrogen species is removed, the oxidation state of the Sn increases.

**Figure 9:**
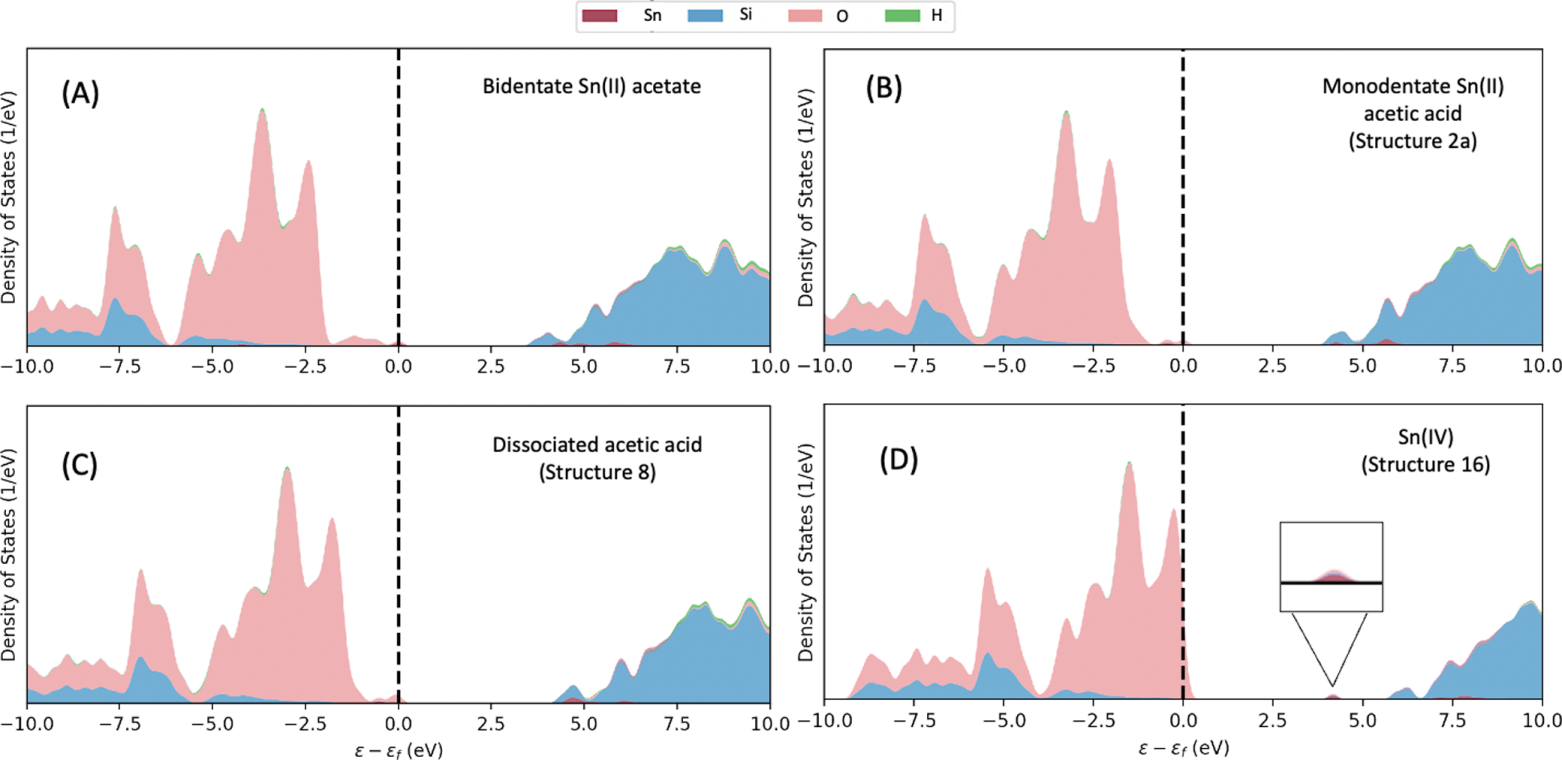
Electronic DOS of different stages in synthesis of Sn-β, as calculated with periodic DFT. (A) Free bidentate Sn(II) acetate in the pore of deAl-β (B) framework-coordinated Sn(II) with acetic acid ligands interacting with the silanol nest (Structure 2a). (C) Framework-coordinated Sn(II) with the acetic acid dis-coordinated (Structure 8). (D) Sn (IV) incorporated into β framework (Structure 16). A key is provided for the DOS interpretation, and a dashed vertical line marks the Fermi level.

Analysis of the density of states (DOS) in Figure 9 for the bidentate Sn(II) acetate in the β pore, coordinated Sn with bound acetic acid ligands (Structure 2a) and coordinated Sn with free acetic acid (Structure 8), shows that the bands associated with Sn remain unchanged throughout. The results indicate that the oxidation state of the Sn(II) is unchanged through the considered steps and is in contrast to the DOS structure of incorporated Sn(IV), which is considered a final product in the SSI process after heating in air flow at elevated temperatures (*i.e.*, 550 °C), as determined by *in situ* XAS studies.^33^ Additionally, the difference in DOS for framework-coordinated Sn(II) and Sn(IV) suggests that interaction with Sn(II) acetate and dissociation of the acetate ligands alone, leaving the framework-coordinated Sn(II), is insufficient to form the final catalyst. The observation is in good agreement with experimental findings, as XAS studies also indicate that Sn remains in a +2 oxidation state until the final step of SSI, where compressed air is introduced into the gas feed.^33^

**Figure 10:**
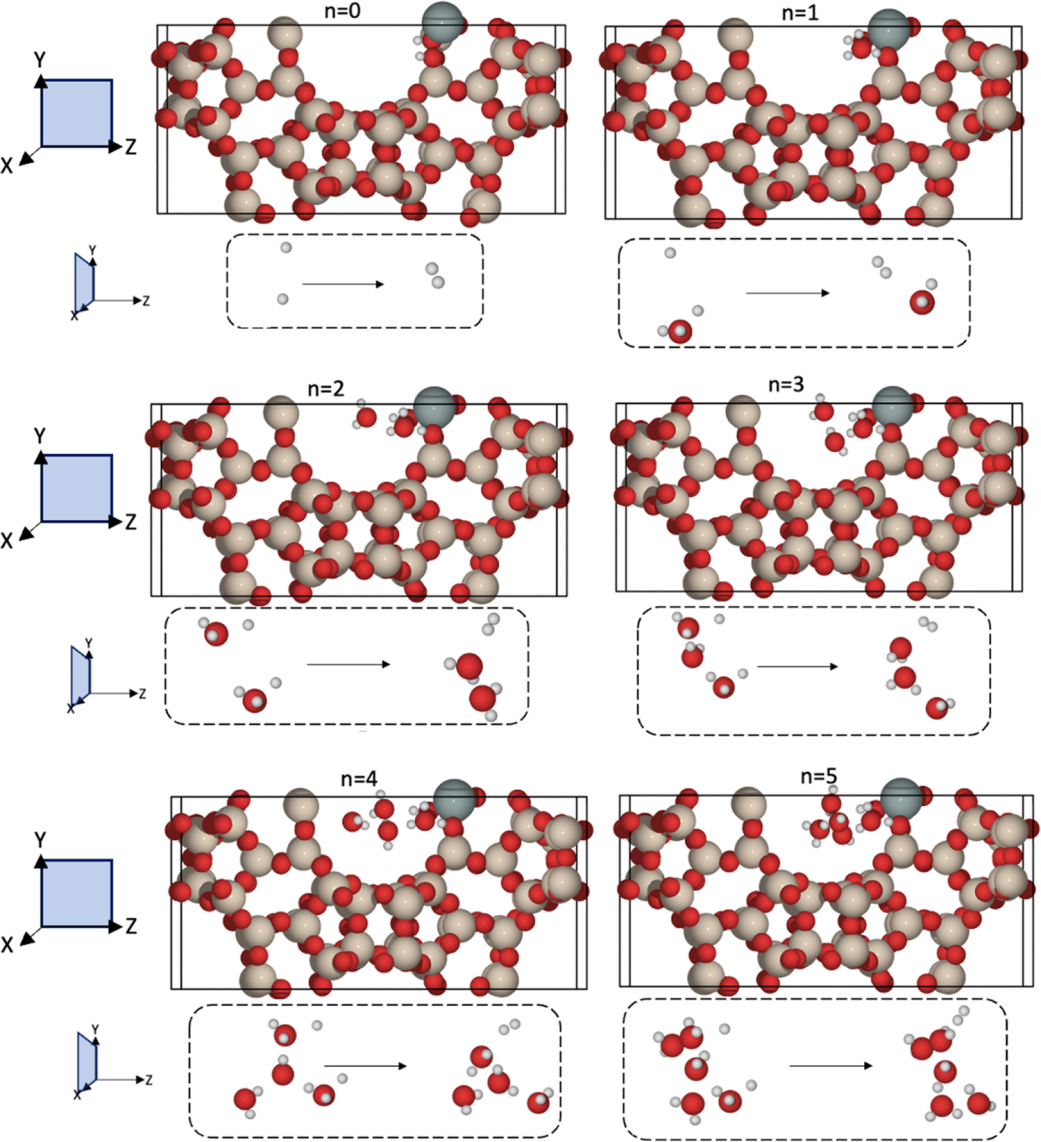
Optimized structures for Sn(II)-β and Sn(IV)-β, with stabilized H_2_O clusters created around the Sn(II) site. Each image is labeled by *n*, which represents the number of H_2_O molecules in the model. The top figures in each case are viewed along the *yz*-plane for the complete simulation cell. The bottom figures, which are viewed along the *xy*-plane, show the change in structure during the conversion of Sn(II) to Sn(IV), with the production of H_2_ mediated by H_2_O (shown in the dashed black boxes). Red, beige, white, gray, and gray atoms represent O, Si, H, C, and Sn, respectively. Results for *n* = 0 and *n* = 3 are reused from ref (33).

**Figure 11:**
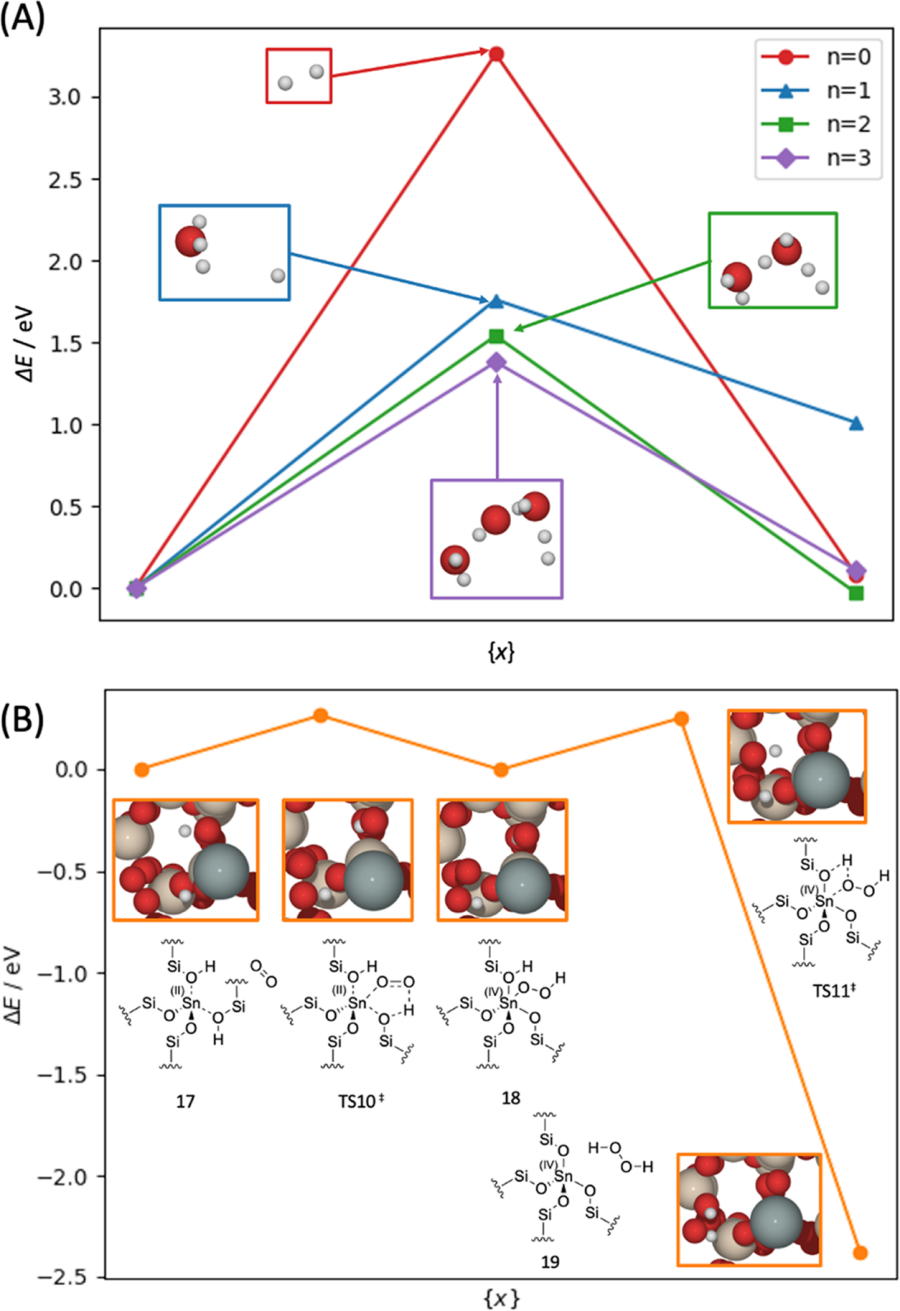
Graph of the reaction energies (Δ*E*) and kinetic barrier (*E*_act_), as calculated with periodic DFT for (A) oxidation of Sn(II) to Sn(IV) depending on the number (*n*) of H_2_O molecules. Insets: transition states for conversion of Sn(II) to Sn(IV), with the production of H_2_ mediated by H_2_O. (B) Oxidation of Sn(II) to Sn(IV) *via* O_2_ producing H_2_O_2_. Red, white, gray, and beige atoms represent O, H, Sn, and Si, respectively. Results in (A) for *n* = 0, 3 are reused from ref (33).

Periodic DFT calculations indicate that the oxidation of Sn(II) to form Sn(IV) is slightly endothermic with a Δ*E* of 0.08 eV. Furthermore, considering first the direct dehydrogenation from Sn(II) in the absence of H_2_O (Figure 10), to form Sn(IV), a very large kinetic barrier of 3.26 eV is calculated with periodic DFT (Figure 11) as reported in previous work;^33^ however, the inclusion of H_2_O reduces the kinetic barrier for oxidation, with the quantity of H_2_O molecules in the framework pore affecting the barrier height. Studies in the literature have examined the role of H_2_O within zeolites, where water molecules facilitate H shuttling in the framework, and have found that H transfer between O atoms around Brønsted acid sites is mediated by water molecules.^88,89^ In that regard, it is interesting to consider the possible role of H_2_O-facilitated dehydrogenation in the oxidation of Sn(II) through shuttling in Lewis acid zeolites, such as Sn-BEA, building on previous studies on Brønsted acid zeolites, where we assume that H_2_O could be introduced to the framework from air during the final stage of SSI or as residual molecules in the β channel after heating.

As the amount of H_2_O in the pore increases, from one to three molecules, the energy barrier for oxidation decreases from 1.76 to 1.54 and 1.38 eV for one, two, and three H_2_O molecules, respectively (Figure 11). Further addition of H_2_O increases the activation energy, where *E*_act_ is calculated as 1.58 and 1.84 eV for four and five H_2_O molecules, respectively (Figure S9). The H_2_O molecules clearly facilitate the oxidation step, due to the improved feasibility of hydrogen shuttling to produce the H_2_ product. QM/MM calculations with MP2 (Table S5) indicate that the charge on the Sn center doubles for oxidation *via* direct dehydrogenation, showing an increase in oxidation state from +2 to +4; a similar increase in charge is seen for the oxidation pathway containing three H_2_O molecules, which suggests the latter, more kinetically viable pathway (with three H_2_O) also leads to the formation of the catalytically active Sn(IV) species.

For the dehydrogenation process necessary to form Sn(IV), the energy as calculated using QM/MM methods (MP2) indicates that direct dehydrogenation of Sn(II)–OH–Si to form Sn(IV)–O–Si in the absence of H_2_O is exothermic, with a dehydrogenation energy (*E*_deH_) of −0.57 eV (Figure S6 and Table S4), in contrast to the energy calculated with periodic DFT (*E*_deH_ of 0.08 eV). The dehydrogenation energy increases to a marginally endothermic value in the presence of three H_2_O molecules (Figure S6, structures 6 and 7), with *E*_deH_ of 0.01 eV (Table S4); the maintenance of low reaction energies and reduced kinetic barriers indicates that a network of H_2_O molecules or hydrogen bonds could facilitate the oxidation of Sn(II) to Sn(IV).

**Figure 12:**
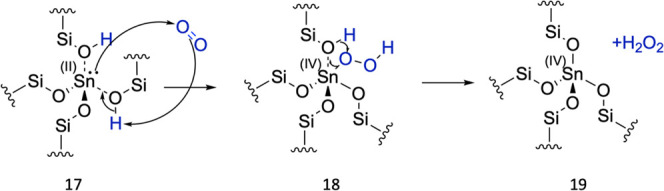
Mechanism for Sn(II) oxidation to Sn(IV) *via* O_2_-facilitated dehydrogenation.

An alternative route for Sn(II) oxidation is through dioxygen (O_2_), where previous studies demonstrate that metal sites in catalytic systems undergo oxidation in this way.^90−92^ It is therefore plausible that O_2_ present in the zeolite pore in the later stages of SSI (550 °C in air flow) can dehydrogenate the framework-bound species and facilitate Sn(II) oxidation. In this mechanism (Figure 12), oxidation occurs in a two-stage process where O_2_ coordinates with Sn(II) and forms a bound −OOH species through H transfer from the Sn–(OH)–Si; subsequently, further H transfer from the other Sn–(OH)–Si group leads to the formation of Sn(IV) and H_2_O_2_, which can later decompose to form H_2_O and O_2_.

Calculations with periodic DFT (Figure 11) indicate that coordination of O_2_ to form bound −OOH (Structure 17 and 18) is marginally exothermic with a Δ*E* of −0.003 eV and *E*_act_ of 0.26 eV. In the second step, the formation of H_2_O_2_ is exothermic with a Δ*E* of −2.38 eV and *E*_act_ of 0.25 eV. The barriers calculated are relatively low, and more than 1 eV lower than those observed in the case of H_2_O-facilitated dehydrogenation. Furthermore, similar to the other oxidation pathways that consider hydrogen shuttling, QM/MM calculations with MP2 (Table S5) indicate that the charge on Sn doubles during dehydrogenation *via* this pathway. Given the greatly reduced kinetic barriers observed for oxidation with O_2_, it is highly plausible that oxidation by O_2_ is the pathway for Sn(II) oxidation to Sn(IV) during this stage of SSI. The simulation results agree with experimental observations, where the water content is similar in the N_2_ and compressed air feeds yet oxidation is observed only upon the introduction of compressed air, which also has a significant concentration of O_2_.^31^

Following the formation of the Sn-β catalyst, successive hydration processes are thought to transform the formed Sn(IV) closed site to an open site, which is considered the more active catalyst speciation; further hydration can yield hexacoordinated Sn centers, which have been observed at the end of heat treatments, and so there is a clear need to understand the role of H_2_O in the formation of the Sn(IV) active site (Figure 10). Studies in the literature suggest that the formation of an open site occurs through the successive adsorption of two H_2_O molecules onto the Sn(IV) site.^93^ Calculations with periodic DFT indicate that the adsorption of one H_2_O onto Sn(IV) is favorable with *E*_ads_ of −0.47 eV, and is also confirmed favorable *via* QM/MM simulations with MP2 (Figure S6, Table S4), where *E*_ads_ of −0.73 eV was calculated; however, the adsorption of a second H_2_O on to Sn(IV) is unfavorable, with a positive *E*_ads_ with MP2 of 0.83 eV. The increase in *E*_ads_ suggests that the formation of open site through H_2_O adsorption may be temperature-dependent, with the presence of open sites more likely at lower temperatures, as suggested by previous studies.^94^

### 3.4 Key Mechanistic Steps

**Figure 13:**
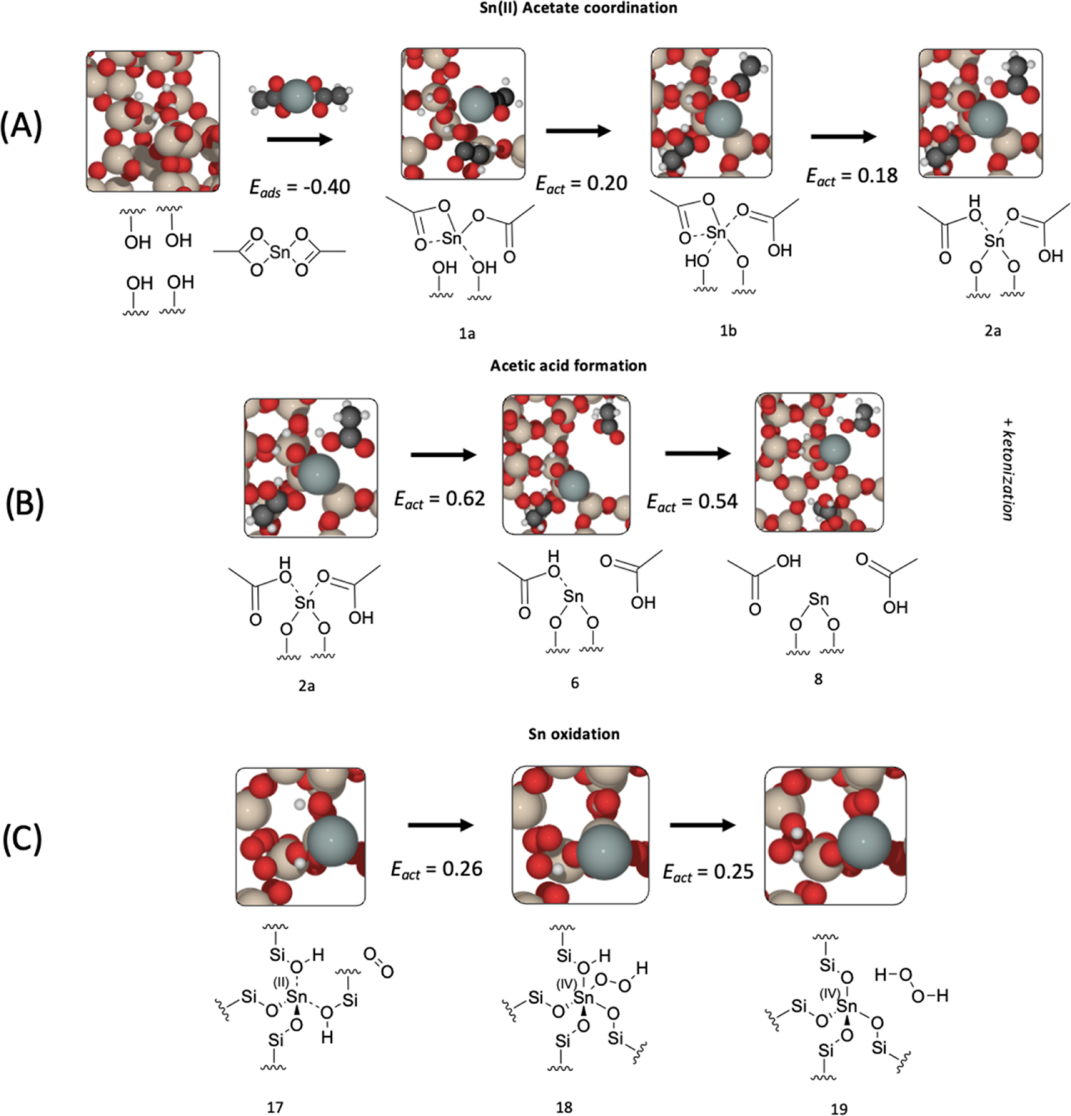
Proposed key stages during the SSI process: (A) Sn coordination, (B) acetic acid removal, and (C) Sn oxidation. All energies are reported in eV. The structure numbers are given under each image. Red, beige, white, black, and gray atoms represent O, Si, H, C, and Sn, respectively.

Considering the role of gas feed species during the oxidation of Sn(II) acetate, along with the change in Sn coordination upon grinding and subsequent formation of acetic acid, it is possible to propose three main stages in SSI for framework coordination of Sn(II), acetate removal, and oxidation of Sn(II) to Sn(IV), as shown in Figure 13.

In Figure 13A, interaction with the framework causes the coordination of Sn(II) acetate to change, converting from bi- to monodentate, which is facilitated by consecutive H transfer from the framework to the Sn(II) acetate *via* low kinetic barriers of 0.20 and 0.18 eV, respectively, forming an acetic acid moiety that stabilizes the monodentate structure by 3.14 eV. The separation of acetic acid from the Sn(II) is facile and readily occurs under the given reaction conditions (Figure 13B), with barriers below 1 eV; furthermore, these Sn-dissociated acetic acid molecules can undergo ketonization to form CO_2_ and acetone, as observed in experimental studies, and calculations indicate that interaction with the Lewis acid Sn site facilitates the reaction by reducing the kinetic barrier from 3.86 to 1.06 eV.

Following the release of the acetic acid moieties, the framework-coordinated Sn center must undergo oxidation from Sn(II) to Sn(IV) to form the active catalyst (Figure 13C). The kinetic barrier for oxidation *via* direct dehydrogenation is calculated to be high, at 3.24 eV, but a network of H_2_O molecules can facilitate dehydrogenation with a reduced barrier of 1.38 eV with 3 H_2_O molecules. Alternatively, the dehydrogenation process may be facilitated by O_2_*via* a two-step process; in this pathway, O_2_ coordinates with the Sn(II) and abstracts each H atom consecutively, as opposed to the concerted mechanism with H_2_O, to form H_2_O_2_. The kinetic barriers for the O_2_-mediated process are greatly alleviated in comparison to the H_2_O-facilitated reaction, with small values of 0.25 and 0.26 eV for the respective steps.

## 4 Conclusions

Computational simulation *via* periodic DFT and embedded-cluster QM/MM have been used to investigate the mechanistic steps of Sn-β synthesis *via* Solid-State Incorporation (SSI). Initially, the bidentate Sn(II) acetate precursor must transform into a monodentate structure when interacting with the framework, which occurs *via* low kinetic barriers of 0.20 and 0.18 eV. Simultaneously, H transfer from the β framework onto the acetate ligand forms a bound acetic acid, which is significantly more stable than monodentate acetate. The release of the acetic acid from the Sn center occurs with an energy barrier below 1 eV, though the connectivity between the Sn and the acetic acid ligand does influence the process; [Sn(II)–(OH)–R] has a smaller kinetic barrier than [Sn(II)–O–R] species for structure 2a, with activation energies of 0.66 and 0.72 eV, respectively. Further periodic DFT simulations show that lower dissociation barriers exist for structure 2b, which is a conformer of 2a, with [Sn(II)–(OH)–R] and [Sn(II)–O–R] having kinetic barriers of 0.00 and 0.03 eV, respectively. Conversion between structures 2a and 2b is endothermic (Δ*E* = 0.46 eV), but considered accessible under reaction conditions, and provides a pathway to the formation of free acetic acid molecules given the low kinetic barriers for acetic acid ligand dissociation for both structures.

Experimental studies^33^ highlight the production of CO_2_ and acetone, rather than acetic acid, and thus simulations were performed to consider ketonization of the acetic acid. The ketonization of gas-phase acetic acid to form acetone, CO_2_, and H_2_O has a kinetic barrier of 3.84 eV, while ketonization over the Lewis acid Sn center has a significantly lower kinetic barrier of 1.06 eV as calculated with periodic DFT. Thus, the ketonization is concluded to be catalyzed by the Sn centers.

For the framework-coordinated Sn species, analysis of the electronic density of states (DOS) indicates that the oxidation state of Sn remains +2 after acetic acid is released, and additional steps must be required to fully incorporate Sn into the β framework and form the active Sn(IV) catalyst. Calculations show that oxidation of Sn(II) to Sn(IV) *via* direct dehydrogenation has a high kinetic barrier of 3.26 eV, but this can be reduced *via* mediated oxidation processes. Two facilitated oxidation processes were considered: H shuttling *via* H_2_O molecules, which is a well-established phenomenon within zeolites, and also O_2_-facilitated dehydrogenation. For the H_2_O-mediated process, a network of three H_2_O molecules lowers the kinetic barrier for Sn oxidation to 1.38 eV with periodic DFT, and QM/MM calculations indicate favorable interaction of H_2_O and a closed Sn(IV) site, which is known to form an open site a possible state for the active catalyst. However, the O_2_-mediated approach indicates an energetically more favorable path for Sn oxidation, where O_2_ forms H_2_O_2_ in a two-step process, with kinetic barriers of 0.25 and 0.26 eV, respectively. Further consideration of the effect of H_2_O and O_2_ in mechanistic models, and experimental testing, will be valuable next steps.

## Data Availability

All periodic DFT structures calculated in this work have been uploaded to the NOMAD repository at DOIs: 10.17172/NOMAD/2022.09.02-2 and 10.17172/NOMAD/2023.09.05-1.

## Supporting Information Available

Information on calculations settings along with energetics and electronic structure analysis for steps in the reaction profile (PDF)

## References

[ref1] Sheldon R. A.; Arens I.; Hanefeld U. Chemistry and Catalysis; WileyVCH: Weinheim, 2007.

[ref2] Corma A.; Nemeth L. T.; Renz M.; Valencia S. Sn-Zeolite Beta as a Heterogeneous Chemoselective Catalyst for Baeyer–Villiger Oxidations. Nature 2001, 412, 423–425. 10.1038/35086546. 11473313

[ref3] Corma A.; Domine M. E.; Nemeth L.; Valencia S. Al-Free Sn-Beta Zeolite as a Catalyst for the Selective Reduction of Carbonyl Compounds (Meerwein–Ponndorf–Verley Reaction). J. Am. Chem. Soc. 2002, 124, 3194–3195. 10.1021/ja012297m. 11916388

[ref4] Otomo R.; Yokoi T.; Kondo J. N.; Tatsumi T. Dealuminated Beta Zeolite as Effective Bifunctional Catalyst for Direct Transformation of Glucose to 5-Hydroxymethylfurfural. Appl. Catal., A 2014, 470, 318–326. 10.1016/j.apcata.2013.11.012.

[ref5] Renz M.; Blasco T.; Corma A.; Fornés V.; Jensen R.; Nemeth L. Selective and Shape-Selective Baeyer–Villiger Oxidations of Aromatic Aldehydes and Cyclic Ketones with Sn-Beta Zeolites and H2O2. Chem. - Eur. J. 2002, 8, 4708–4717. 10.1002/1521-3765(20021018)8:20<4708::AID-CHEM4708≥3.0.CO;2-U. 12561111

[ref6] Boronat M.; Corma A.; Renz M.; Sastre G.; Viruela P. M. A Multisite Molecular Mechanism for Baeyer-Villiger Oxidations on Solid Catalysts Using Environmentally Friendly H2O2 as Oxidant. Chem. - Eur. J. 2005, 11, 6905–6915. 10.1002/chem.200500184. 16163761

[ref7] Peeters E.; Pomalaza G.; Khalil I.; Detaille A.; Debecker D. P.; Douvalis A. P.; Dusselier M.; Sels B. F. Highly Dispersed Sn-Beta Zeolites as Active Catalysts for Baeyer–Villiger Oxidation: The Role of Mobile, *In Situ* Sn(II)O Species in Solid-State Stannation. ACS Catal. 2021, 11, 5984–5998. 10.1021/acscatal.1c00435.

[ref8] Primo A.; Garcia H. Zeolites as Catalysts in Oil Refining. Chem. Soc. Rev. 2014, 43, 7548–7561. 10.1039/C3CS60394F. 24671148

[ref9] Mal N. K.; Ramaswamy A. V. Synthesis and Catalytic Properties of Large-Pore Sn-β and Al-Free Sn-β Molecular Sieves. Chem. Commun. 1997, 5, 425–426. 10.1039/a607038h.

[ref10] Yakabi K.; Mathieux T.; Milne K.; López-Vidal E. M.; Buchard A.; Hammond C. Continuous Production of Biorenewable, Polymer-Grade Lactone Monomers through Sn-β-Catalyzed Baeyer-Villiger Oxidation with H _2_ O _2_. ChemSusChem 2017, 10, 3652–3659. 10.1002/cssc.201701298. 28804968 PMC5708276

[ref11] Lewis J. D.; Van de Vyver S.; Crisci A. J.; Gunther W. R.; Michaelis V. K.; Griffin R. G.; Román-Leshkov Y. A Continuous Flow Strategy for the Coupled Transfer Hydrogenation and Etherification of 5-(Hydroxymethyl)Furfural Using Lewis Acid Zeolites. ChemSusChem 2014, 7, 2255–2265. 10.1002/cssc.201402100. 25045144

[ref12] Padovan D.; Al-Nayili A.; Hammond C. Bifunctional Lewis and Brønsted Acidic Zeolites Permit the Continuous Production of Bio-Renewable Furanic Ethers. Green Chem. 2017, 19, 2846–2854. 10.1039/C7GC00160F.

[ref13] Gilkey M. J.; Xu B. Heterogeneous Catalytic Transfer Hydrogenation as an Effective Pathway in Biomass Upgrading. ACS Catal. 2016, 6, 1420–1436. 10.1021/acscatal.5b02171.

[ref14] Taarning E.; Saravanamurugan S.; Spangsberg Holm M.; Xiong J.; West R. M.; Christensen C. H. Zeolite-Catalyzed Isomerization of Triose Sugars. ChemSusChem 2009, 2, 625–627. 10.1002/cssc.200900099. 19562790

[ref15] Bermejo-Deval R.; Assary R. S.; Nikolla E.; Moliner M.; Román-Leshkov Y.; Hwang S.-J.; Palsdottir A.; Silverman D.; Lobo R. F.; Curtiss L. A.; Davis M. E. Metalloenzyme-like Catalyzed Isomerizations of Sugars by Lewis Acid Zeolites. Proc. Natl. Acad. Sci. U.S.A. 2012, 109, 9727–9732. 10.1073/pnas.1206708109. 22665778 PMC3382492

[ref16] Román-Leshkov Y.; Moliner M.; Labinger J. A.; Davis M. E. Mechanism of Glucose Isomerization Using a Solid Lewis Acid Catalyst in Water. Angew. Chem., Int. Ed. 2010, 49, 8954–8957. 10.1002/anie.201004689. 20963742

[ref17] Moliner M.; Román-Leshkov Y.; Davis M. E. Tin-Containing Zeolites Are Highly Active Catalysts for the Isomerization of Glucose in Water. Proc. Natl. Acad. Sci. U.S.A. 2010, 107, 6164–6168. 10.1073/pnas.1002358107. 20308577 PMC2852018

[ref18] Holm M. S.; Saravanamurugan S.; Taarning E. Conversion of Sugars to Lactic Acid Derivatives Using Heterogeneous Zeotype Catalysts. Science 2010, 328, 602–605. 10.1126/science.1183990. 20431010

[ref19] Zhang Y.; Luo H.; Zhao X.; Zhu L.; Miao G.; Wang H.; Li S.; Kong L. Continuous Conversion of Glucose into Methyl Lactate over the Sn-Beta Zeolite: Catalytic Performance and Activity Insight. Ind. Eng. Chem. Res. 2020, 59, 17365–17372. 10.1021/acs.iecr.0c01770.

[ref20] Jin J.; Ye X.; Li Y.; Wang Y.; Li L.; Gu J.; Zhao W.; Shi J. Synthesis of Mesoporous Beta and Sn-Beta Zeolites and Their Catalytic Performances. Dalton Trans. 2014, 43, 8196–8204. 10.1039/C4DT00567H. 24777171

[ref21] Tang B.; Dai W.; Sun X.; Guan N.; Li L.; Hunger M. A Procedure for the Preparation of Ti-Beta Zeolites for Catalytic Epoxidation with Hydrogen Peroxide. Green Chem. 2014, 16, 2281–2291. 10.1039/C3GC42534G.

[ref22] Corma A. State of the Art and Future Challenges of Zeolites as Catalysts. J. Catal. 2003, 216, 298–312. 10.1016/S0021-9517(02)00132-X.

[ref23] Bates J. S.; Bukowski B. C.; Harris J. W.; Greeley J.; Gounder R. Distinct Catalytic Reactivity of Sn Substituted in Framework Locations and at Defect Grain Boundaries in Sn-Zeolites. ACS Catal. 2019, 9, 6146–6168. 10.1021/acscatal.9b01123.

[ref24] Dijkmans J.; Dusselier M.; Janssens W.; Trekels M.; Vantomme A.; Breynaert E.; Kirschhock C.; Sels B. F. An Inner-/Outer-Sphere Stabilized Sn Active Site in β-Zeolite: Spectroscopic Evidence and Kinetic Consequences. ACS Catal. 2016, 6, 31–46. 10.1021/acscatal.5b01822.

[ref25] Wolf P.; Valla M.; Núñez-Zarur F.; Comas-Vives A.; Rossini A. J.; Firth C.; Kallas H.; Lesage A.; Emsley L.; Copéret C.; et al. Correlating Synthetic Methods, Morphology, Atomic-Level Structure, and Catalytic Activity of Sn-β Catalysts. ACS Catal. 2016, 6, 4047–4063. 10.1021/acscatal.6b00114.

[ref26] Josephson T. R.; Jenness G. R.; Vlachos D. G.; Caratzoulas S. Distribution of Open Sites in Sn-Beta Zeolite. Microporous Mesoporous Mater. 2017, 245, 45–50. 10.1016/j.micromeso.2017.02.065.

[ref27] Wolf P.; Hammond C.; Conrad S.; Hermans I. Post-Synthetic Preparation of Sn-, Ti- and Zr-Beta: A Facile Route to Water Tolerant, Highly Active Lewis Acidic Zeolites. Dalton Trans. 2014, 43, 4514–4519. 10.1039/c3dt52972j. 24407516

[ref28] Wu P.; Komatsu T.; Yashima T.; Nakata S.; Shouji H. Modification of Mordenite Acidity by Isomorphous Substitution of Trivalent Cations in the Framework Sites Using the Atom-Planting Method. Microporous Mater. 1997, 12, 25–37. 10.1016/S0927-6513(97)00056-4.

[ref29] Rigutto M. S.; de Ruiter R.; Niederer J. P. M.; van Bekkum H. Titanium-Containing Large Pore Molecular Sieves from Boron-Beta: Preparation, Characterization and Catalysis. In Studies in Surface Science and Catalysis; Elsevier, 1994; Vol. 84, pp 2245–2252.

[ref30] Blasco T.; Camblor M. A.; Corma A.; Esteve P.; Guil J. M.; Martínez A.; Perdigón-Melón J. A.; Valencia S. Direct Synthesis and Characterization of Hydrophobic Aluminum-Free Ti–Beta Zeolite. J. Phys. Chem. B 1998, 102, 75–88. 10.1021/jp973288w.

[ref31] Hammond C.; Conrad S.; Hermans I. Simple and Scalable Preparation of Highly Active Lewis Acidic Sn-β. Angew. Chem., Int. Ed. 2012, 51, 11736–11739. 10.1002/anie.201206193. 23042488

[ref32] Hammond C.; Padovan D.; Al-Nayili A.; Wells; Peter P.; Gibson E. K.; Dimitratos N. Identification of Active and Spectator Sn Sites in Sn-β Following Solid-State Stannation, and Consequences for Lewis Acid Catalysis. ChemCatChem 2015, 7, 3322–3331. 10.1002/cctc.201500545. 26583051 PMC4641460

[ref33] Navar R.; Tarantino G.; Beynon O. T.; Padovan D.; Botti L.; Gibson E. K.; Wells P. P.; Owens A.; Kondrat S.; Logsdail A. J.; Hammond C. Tracking the Solid-State Incorporation of Sn into the Framework of Dealuminated Zeolite Beta, and Consequences for Catalyst Design. J. Mater. Chem. A 2022, 10, 22025–22041. 10.1039/D2TA03837D.

[ref34] Padovan D.; Botti L.; Hammond C. Active Site Hydration Governs the Stability of Sn-Beta during Continuous Glucose Conversion. ACS Catal. 2018, 8, 7131–7140. 10.1021/acscatal.8b01759.

[ref35] Botti L.; Navar R.; Tolborg S.; Martínez-Espín J. S.; Hammond C. High-Productivity Continuous Conversion of Glucose to α-Hydroxy Esters over Postsynthetic and Hydrothermal Sn-Beta Catalysts. ACS Sustainable Chem. Eng. 2022, 10, 4391–4403. 10.1021/acssuschemeng.1c06989. 35433137 PMC9007564

[ref36] Botti L.; Navar R.; Tolborg S.; Martinez-Espin J. S.; Padovan D.; Taarning E.; Hammond C. Influence of Composition and Preparation Method on the Continuous Performance of Sn-Beta for Glucose-Fructose Isomerisation. Top. Catal. 2019, 62, 1178–1191. 10.1007/s11244-018-1078-z.

[ref37] Yang G.; Pidko E. A.; Hensen E. J. M. Structure, Stability, and Lewis Acidity of Mono and Double Ti, Zr, and Sn Framework Substitutions in BEA Zeolites: A Periodic Density Functional Theory Study. J. Phys. Chem. C 2013, 117, 3976–3986. 10.1021/jp310433r.

[ref38] Boronat M.; Concepcion P.; Corma A.; Renz M.; Valencia S. Determination of the Catalytically Active Oxidation Lewis Acid Sites in Sn-Beta Zeolites, and Their Optimisation by the Combination of Theoretical and Experimental Studies. J. Catal. 2005, 234, 111–118. 10.1016/j.jcat.2005.05.023.

[ref39] Kulkarni B. S.; Krishnamurty S.; Pal S. Probing Lewis Acidity and Reactivity of Sn- and Ti-Beta Zeolite Using Industrially Important Moieties: A Periodic Density Functional Study. J. Mol. Catal. A: Chem. 2010, 329, 36–43. 10.1016/j.molcata.2010.06.014.

[ref40] Shetty S.; Pal S.; Kanhere D. G.; Goursot A. Structural, Electronic, and Bonding Properties of Zeolite Sn-Beta: A Periodic Density Functional Theory Study. Chem. - Eur. J. 2006, 12, 518–523. 10.1002/chem.200500487. 16187377

[ref41] Petkov P. St.; Aleksandrov H. A.; Valtchev V.; Vayssilov G. N. Framework Stability of Heteroatom-Substituted Forms of Extra-Large-Pore Ge-Silicate Molecular Sieves: The Case of ITQ-44. Chem. Mater. 2012, 24, 2509–2518. 10.1021/cm300861e.

[ref42] Shetty S.; Kulkarni B. S.; Kanhere D. G.; Goursot A.; Pal S. A Comparative Study of Structural, Acidic and Hydrophilic Properties of Sn–BEA with Ti–BEA Using Periodic Density Functional Theory. J. Phys. Chem. B 2008, 112, 2573–2579. 10.1021/jp709846s. 18269277

[ref43] Yang G.; Zhou L.; Han X. Lewis and Brönsted Acidic Sites in M4+-Doped Zeolites (M = Ti, Zr, Ge, Sn, Pb) as Well as Interactions with Probe Molecules: A DFT Study. J. Mol. Catal. A: Chem. 2012, 363–364, 371–379. 10.1016/j.molcata.2012.07.013.

[ref44] O’Malley A. J.; Logsdail A. J.; Sokol A. A.; Catlow C. R. A. Modelling Metal Centres, Acid Sites and Reaction Mechanisms in Microporous Catalysts. Faraday Discuss. 2016, 188, 235–255. 10.1039/C6FD00010J. 27136967

[ref45] Piccini G.; Alessio M.; Sauer J. Ab Initio Calculation of Rate Constants for Molecule-Surface Reactions with Chemical Accuracy. Angew. Chem., Int. Ed. 2016, 55, 5235–5237. 10.1002/anie.201601534. PMC4834608 27008460

[ref46] Montejo-Valencia B. D.; Curet-Arana M. C. DFT Study of the Lewis Acidities and Relative Hydrothermal Stabilities of BEC and BEA Zeolites Substituted with Ti, Sn, and Ge. J. Phys. Chem. C 2015, 119, 4148–4157. 10.1021/jp512269s.

[ref47] Montejo-Valencia B. D.; Salcedo-Pérez J. L.; Curet-Arana M. C. DFT Study of Closed and Open Sites of BEA, FAU, MFI, and BEC Zeolites Substituted with Tin and Titanium. J. Phys. Chem. C 2016, 120, 2176–2186. 10.1021/acs.jpcc.5b09815.

[ref48] Zhao R.; Zhao Z.; Li S.; Zhang W. Insights into the Correlation of Aluminum Distribution and Brönsted Acidity in H-Beta Zeolites from Solid-State NMR Spectroscopy and DFT Calculations. J. Phys. Chem. Lett. 2017, 8, 2323–2327. 10.1021/acs.jpclett.7b00711. 28488869

[ref49] Wang S.; Guo S.; Luo Y.; Qin Z.; Chen Y.; Dong M.; Li J.; Fan W.; Wang J. Direct Synthesis of Acetic Acid from Carbon Dioxide and Methane over Cu-Modulated BEA, MFI, MOR and TON Zeolites: A Density Functional Theory Study. Catal. Sci. Technol. 2019, 9, 6613–6626. 10.1039/C9CY01803D.

[ref50] Margarit V. J.; Gallego E. M.; Paris C.; Boronat M.; Moliner M.; Corma A. Production of Aromatics from Biomass by Computer-Aided Selection of the Zeolite Catalyst. Green Chem. 2020, 22, 5123–5131. 10.1039/D0GC01031F.

[ref51] Blum V.; Gehrke R.; Hanke F.; Havu P.; Havu V.; Ren X.; Reuter K.; Scheffler M. Ab Initio Molecular Simulations with Numeric Atom-Centered Orbitals. Comput. Phys. Commun. 2009, 180, 2175–2196. 10.1016/j.cpc.2009.06.022.

[ref52] Perdew J. P.; Ruzsinszky A.; Csonka G. I.; Vydrov O. A.; Scuseria G. E.; Constantin L. A.; Zhou X.; Burke K. Restoring the Density-Gradient Expansion for Exchange in Solids and Surfaces. Phys. Rev. Lett. 2008, 100, 136406 10.1103/PhysRevLett.100.136406. 18517979

[ref53] Tkatchenko A.; Scheffler M. Accurate Molecular Van Der Waals Interactions from Ground-State Electron Density and Free-Atom Reference Data. Phys. Rev. Lett. 2009, 102, 073005 10.1103/PhysRevLett.102.073005. 19257665

[ref54] Monkhorst H. J.; Pack J. D. Special Points for Brillouin-Zone Integrations. Phys. Rev. B 1976, 13, 5188–5192. 10.1103/PhysRevB.13.5188.

[ref55] van Lenthe E.; Baerends E. J.; Snijders J. G. Relativistic Total Energy Using Regular Approximations. J. Chem. Phys. 1994, 101, 9783–9792. 10.1063/1.467943.

[ref56] Larsen A. H.; Mortensen J. J.; Blomqvist J.; Castelli I. E.; Christensen R.; Dułak M.; Friis J.; Groves M. N.; Hammer B.; Hargus C.; et al. The Atomic Simulation Environment—a Python Library for Working with Atoms. J. Phys.: Condens. Matter 2017, 29, 273002 10.1088/1361-648X/aa680e. 28323250

[ref57] Newsam J. M.; Treacy M. M. J.; Koetsier W. T.; De Gruyter C. B. Structural Characterization of Zeolite Beta. Proc. R. Soc. London, Ser. A 1988, 420, 375–405. 10.1098/rspa.1988.0131.

[ref58] Broyden C. G. The Convergence of a Class of Double-Rank Minimization Algorithms 1. General Considerations. IMA J. Appl. Math. 1970, 6, 76–90. 10.1093/imamat/6.1.76.

[ref59] Fletcher R. A New Approach to Variable Metric Algorithms. Comput. J. 1970, 13, 317–322. 10.1093/comjnl/13.3.317.

[ref60] Goldfarb D. A Family of Variable-Metric Methods Derived by Variational Means. Math. Comput. 1970, 24, 23 10.1090/S0025-5718-1970-0258249-6.

[ref61] Shanno D. F. Conditioning of Quasi-Newton Methods for Function Minimization. Math. Comput. 1970, 24, 647 10.1090/S0025-5718-1970-0274029-X.

[ref62] Torres J. A. G.; Jennings P. C.; Hansen M. H.; Boes J. R.; Bligaard T. Low-Scaling Algorithm for Nudged Elastic Band Calculations Using a Surrogate Machine Learning Model. Phys. Rev. Lett. 2019, 122, 156001 10.1103/PhysRevLett.122.156001. 31050513

[ref63] Lu Y.; Farrow M. R.; Fayon P.; Logsdail A. J.; Sokol A. A.; Catlow C. R. A.; Sherwood P.; Keal T. W. Open-Source, Python-Based Redevelopment of the ChemShell Multiscale QM/MM Environment. J. Chem. Theory Comput. 2019, 15, 1317–1328. 10.1021/acs.jctc.8b01036. 30511845

[ref64] Nastase S. A. F.; O’Malley A. J.; Catlow C. R. A.; Logsdail A. J. Computational QM/MM Investigation of the Adsorption of MTH Active Species in H-Y and H-ZSM-5. Phys. Chem. Chem. Phys. 2019, 21, 2639–2650. 10.1039/C8CP06736H. 30657492

[ref65] Nastase S. A. F.; Logsdail A. J.; Catlow C. R. A. QM/MM Study of the Reactivity of Zeolite Bound Methoxy and Carbene Groups. Phys. Chem. Chem. Phys. 2021, 23, 17634–17644. 10.1039/D1CP02535J. 34369957

[ref66] Sherwood P.; de Vries A. H.; Collins S. J.; Greatbanks S. P.; Burton N. A.; Vincent M. A.; Hillier I. H. Computer Simulation of Zeolite Structure and Reactivity Using Embedded Cluster Methods. Faraday Discuss. 1997, 106, 79–92. 10.1039/a701790a.

[ref67] Adamo C.; Barone V. Toward Reliable Density Functional Methods without Adjustable Parameters: The PBE0 Model. J. Chem. Phys. 1999, 110, 6158–6170. 10.1063/1.478522.

[ref68] Ren X.; Rinke P.; Blum V.; Wieferink J.; Tkatchenko A.; Sanfilippo A.; Reuter K.; Scheffler M. Resolution-of-Identity Approach to Hartree–Fock, Hybrid Density Functionals, RPA, MP2 and *GW* with Numeric Atom-Centered Orbital Basis Functions. New J. Phys. 2012, 14, 053020 10.1088/1367-2630/14/5/053020.

[ref69] Gale J. D. GULP: A Computer Program for the Symmetry-Adapted Simulation of Solids. Faraday Trans. 1997, 93, 629–637. 10.1039/a606455h.

[ref70] Hill J. R.; Sauer J. Molecular Mechanics Potential for Silica and Zeolite Catalysts Based on Ab Initio Calculations. 1. Dense and Microporous Silica. J. Phys. Chem. A 1994, 98, 1238–1244. 10.1021/j100055a032.

[ref71] Hill J.-R.; Sauer J. Molecular Mechanics Potential for Silica and Zeolite Catalysts Based on Ab Initio Calculations. 2. Aluminosilicates. J. Phys. Chem. A 1995, 99, 9536–9550. 10.1021/j100023a036.

[ref72] Kästner J.; Carr J. M.; Keal T. W.; Thiel W.; Wander A.; Sherwood P. DL-FIND: An Open-Source Geometry Optimizer for Atomistic Simulations. J. Phys. Chem. A 2009, 113, 11856–11865. 10.1021/jp9028968. 19639948

[ref73] Joshi H.; Ochoa-Hernández C.; Nürenberg E.; Kang L.; Wang F. R.; Weidenthaler C.; Schmidt W.; Schüth F. Insights into the Mechanochemical Synthesis of Sn-β: Solid-State Metal Incorporation in Beta Zeolite. Microporous Mesoporous Mater. 2020, 309, 110566 10.1016/j.micromeso.2020.110566.

[ref74] Bare S. R.; Kelly S. D.; Sinkler W.; Low J. J.; Modica F. S.; Valencia S.; Corma A.; Nemeth L. T. Uniform Catalytic Site in Sn-β-Zeolite Determined Using X-Ray Absorption Fine Structure. J. Am. Chem. Soc. 2005, 127, 12924–12932. 10.1021/ja052543k. 16159286

[ref75] Wolf P.; Valla M.; Rossini A. J.; Comas-Vives A.; Núñez-Zarur F.; Malaman B.; Lesage A.; Emsley L.; Copéret C.; Hermans I. NMR Signatures of the Active Sites in Sn-β Zeolite. Angew. Chem. 2014, 126, 10343–10347. 10.1002/ange.201403905. 25079352

[ref76] Smart B. A.; Griffiths L. E.; Pulham C. R.; Robertson H. E.; Mitzel N. W.; Rankin D. W. H. Molecular Structure of Tin(II) Acetate as Determined in the Gas Phase by Electron Diffraction and Ab Initio Calculations. J. Chem. Soc., Dalton Trans. 1997, 9, 1565–1570. 10.1039/a608356k.

[ref77] Settimo L.; Bellman K.; Knegtel R. M. A. Comparison of the Accuracy of Experimental and Predicted PKa Values of Basic and Acidic Compounds. Pharm. Res. 2014, 31, 1082–1095. 10.1007/s11095-013-1232-z. 24249037

[ref78] Gabrienko A. A.; Danilova I. G.; Arzumanov S. S.; Toktarev A. V.; Freude D.; Stepanov A. G. Strong Acidity of Silanol Groups of Zeolite Beta: Evidence from the Studies by IR Spectroscopy of Adsorbed CO and 1H MAS NMR. Microporous Mesoporous Mater. 2010, 131, 210–216. 10.1016/j.micromeso.2009.12.025.

[ref79] Dalstein L.; Potapova E.; Tyrode E. The Elusive Silica/Water Interface: Isolated Silanols under Water as Revealed by Vibrational Sum Frequency Spectroscopy. Phys. Chem. Chem. Phys. 2017, 19, 10343–10349. 10.1039/C7CP01507K. 28379259

[ref80] Ong S.; Zhao X.; Eisenthal K. B. Polarization of Water Molecules at a Charged Interface: Second Harmonic Studies of the Silica/Water Interface. Chem. Phys. Lett. 1992, 191, 327–335. 10.1016/0009-2614(92)85309-X.

[ref81] Dib E.; Costa I. M.; Vayssilov G. N.; Aleksandrov H. A.; Mintova S. Complex H-Bonded Silanol Network in Zeolites Revealed by IR and NMR Spectroscopy Combined with DFT Calculations. J. Mater. Chem. A 2021, 9, 27347–27352. 10.1039/D1TA06908J.

[ref82] Pfeiffer-Laplaud M.; Costa D.; Tielens F.; Gaigeot M.-P.; Sulpizi M. Bimodal Acidity at the Amorphous Silica/Water Interface. J. Phys. Chem. C 2015, 119, 27354–27362. 10.1021/acs.jpcc.5b02854.

[ref83] Busca G. Catalytic Materials Based on Silica and Alumina: Structural Features and Generation of Surface Acidity. Prog. Mater. Sci. 2019, 104, 215–249. 10.1016/j.pmatsci.2019.04.003.

[ref84] Provis J. L. Activating Solution Chemistry for Geopolymers. In Geopolymers; Elsevier, 2009; pp 50–71.

[ref85] Gumidyala A.; Sooknoi T.; Crossley S. Selective Ketonization of Acetic Acid over HZSM-5: The Importance of Acyl Species and the Influence of Water. J. Catal. 2016, 340, 76–84. 10.1016/j.jcat.2016.04.017.

[ref86] Jahangiri H.; Osatiashtiani A.; Ouadi M.; Hornung A.; Lee A. F.; Wilson K. Ga/HZSM-5 Catalysed Acetic Acid Ketonisation for Upgrading of Biomass Pyrolysis Vapours. Catalysts 2019, 9, 841 10.3390/catal9100841.

[ref87] Gomes G. J.; Zalazar M. F.; Lindino C. A.; Scremin F. R.; Bittencourt P. R. S.; Costa M. B.; Peruchena N. M. Adsorption of Acetic Acid and Methanol on H-Beta Zeolite: An Experimental and Theoretical Study. Microporous Mesoporous Mater. 2017, 252, 17–28. 10.1016/j.micromeso.2017.06.008.

[ref88] Ryder J. A.; Chakraborty A. K.; Bell A. T. Density Functional Theory Study of Proton Mobility in Zeolites: Proton Migration and Hydrogen Exchange in ZSM-5. J. Phys. Chem. B 2000, 104, 6998–7011. 10.1021/jp9943427.

[ref89] Liu P.; Mei D. Identifying Free Energy Landscapes of Proton-Transfer Processes between Brønsted Acid Sites and Water Clusters Inside the Zeolite Pores. J. Phys. Chem. C 2020, 124, 22568–22576. 10.1021/acs.jpcc.0c07033.

[ref90] Dinda S.; Genest A.; Rösch N. O _2_ Activation and Catalytic Alcohol Oxidation by Re Complexes with Redox-Active Ligands: A DFT Study of Mechanism. ACS Catal. 2015, 5, 4869–4880. 10.1021/acscatal.5b00509.

[ref91] Toledo A.; Funes-Ardoiz I.; Maseras F.; Albéniz A. C. Palladium-Catalyzed Aerobic Homocoupling of Alkynes: Full Mechanistic Characterization of a More Complex Oxidase-Type Behavior. ACS Catal. 2018, 8, 7495–7506. 10.1021/acscatal.8b01540.

[ref92] Popp B. V.; Wendlandt J. E.; Landis C. R.; Stahl S. S. Reaction of Molecular Oxygen with an NHC-Coordinated Pd0 Complex: Computational Insights and Experimental Implications. Angew. Chem., Int. Ed. 2007, 46, 601–604. 10.1002/anie.200603667. 17154213

[ref93] Sushkevich V. L.; Kots P. A.; Kolyagin Y. G.; Yakimov A. V.; Marikutsa A. V.; Ivanova I. I. Origin of Water-Induced Brønsted Acid Sites in Sn-BEA Zeolites. J. Phys. Chem. C 2019, 123, 5540–5548. 10.1021/acs.jpcc.8b12462.

[ref94] Courtney T. D.; Chang C.-C.; Gorte R. J.; Lobo R. F.; Fan W.; Nikolakis V. Effect of Water Treatment on Sn-BEA Zeolite: Origin of 960 Cm–1 FTIR Peak. Microporous Mesoporous Mater. 2015, 210, 69–76. 10.1016/j.micromeso.2015.02.012.

