# Supplementary material for: Computational Study of the Solid-State Incorporation of Sn(II) Acetate into Zeolite β
Source: J Phys Chem C Nanomater Interfaces. 2023 Sep 15;127(38):19072–87. doi: 10.1021/acs.jpcc.3c02679 (PMC10544035; doi:10.1021/acs.jpcc.3c02679)
Supplement: Supplementary file 1 — jp3c02679_si_001.pdf [file jp3c02679_si_001.pdf]

Supporting Information

Computational Study of the Solid-State Incorporation of Sn(II) Acetate into Zeolite β

*Owain T. Beynon[1], Alun Owens[1], Giulia Tarantino[2], Ceri Hammond[2], Andrew J. Logsdail[1]**

1. Cardiff Catalysis Institute, Cardiff University, Park Place, Cardiff, CF10 3AT, Wales, UK

2. Department of Chemical Engineering, Imperial College London, London, SW7 2AZ, UK

*LogsdailA@cardiff.ac.uk

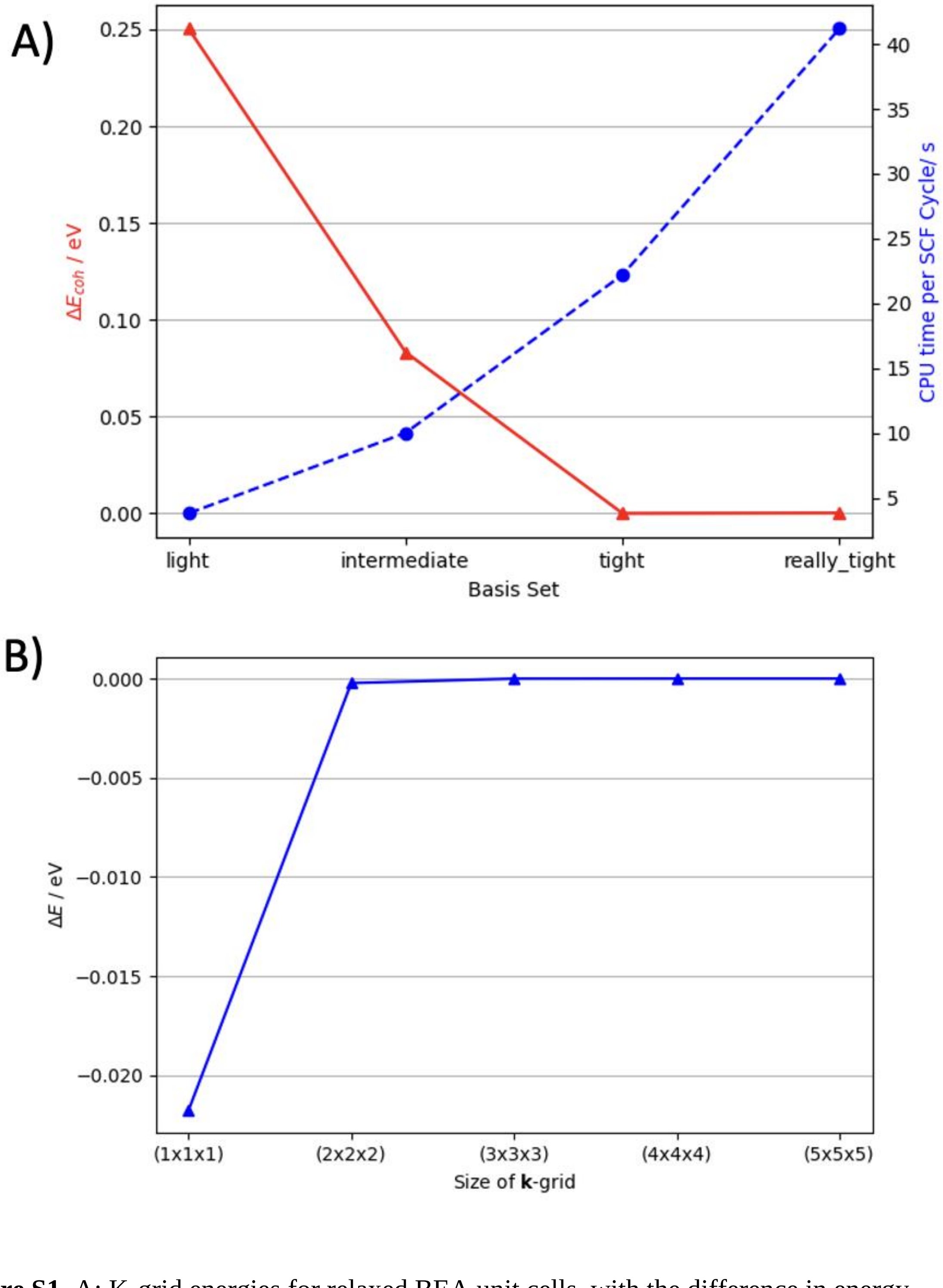

Figure S1. A: K-grid energies for relaxed BEA unit cells, with the difference in energy between the lowest value and all others reported as ΔE/ eV. B: Difference in cohesive energy (ΔE_{coh}/ eV) for forming BEA as a function of basis set size and CPU time per SCF cycle.

Table S1: Comparison of exchange-correlation functionals for the optimisation of the BEA unit cell, with resultant lattice parameters *a*, *b*, and *c* (Å).

	a	***B***	***c***
Experiment[1]	*12.632*	*12.632*	*26.186*
**PW-LDA**	12.372	12.372	26.231
**BLYP**	12.784	12.784	26.843
**PBE**	12.680	12.680	26.644
**PBE + TS**	12.450	12.450	26.484
**PBE + MBD**	12.526	12.526	26.424
**PBEsol**	12.551	12.551	26.450
**PBEsol + TS**	12.467	12.467	26.406
**B3LYP**	12.683	12.683	26.630
**PBE0**	12.587	12.587	26.444
**HF**	12.582	12.582	26.484

A)

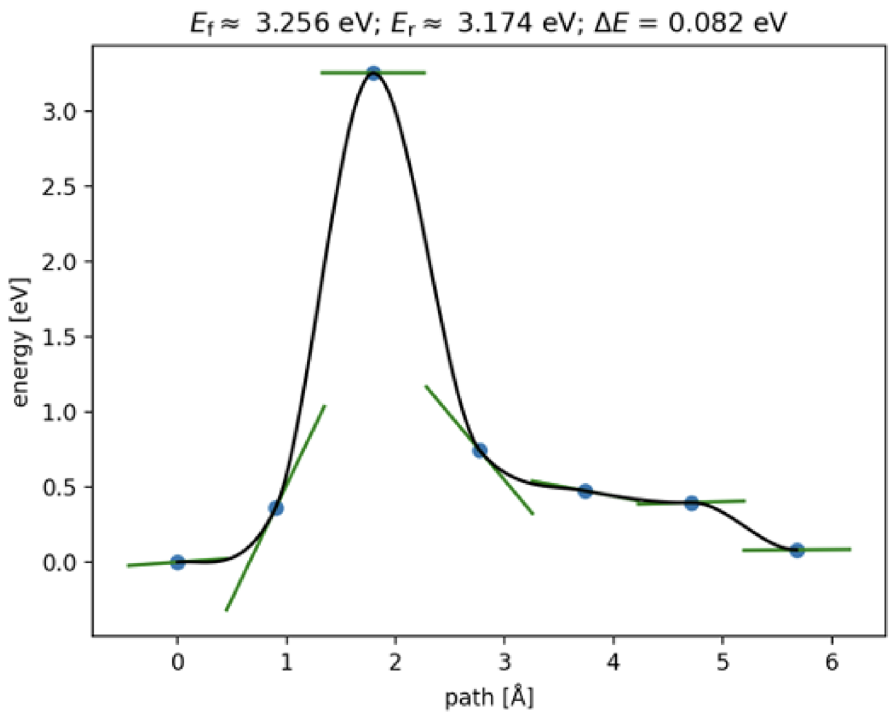

B)

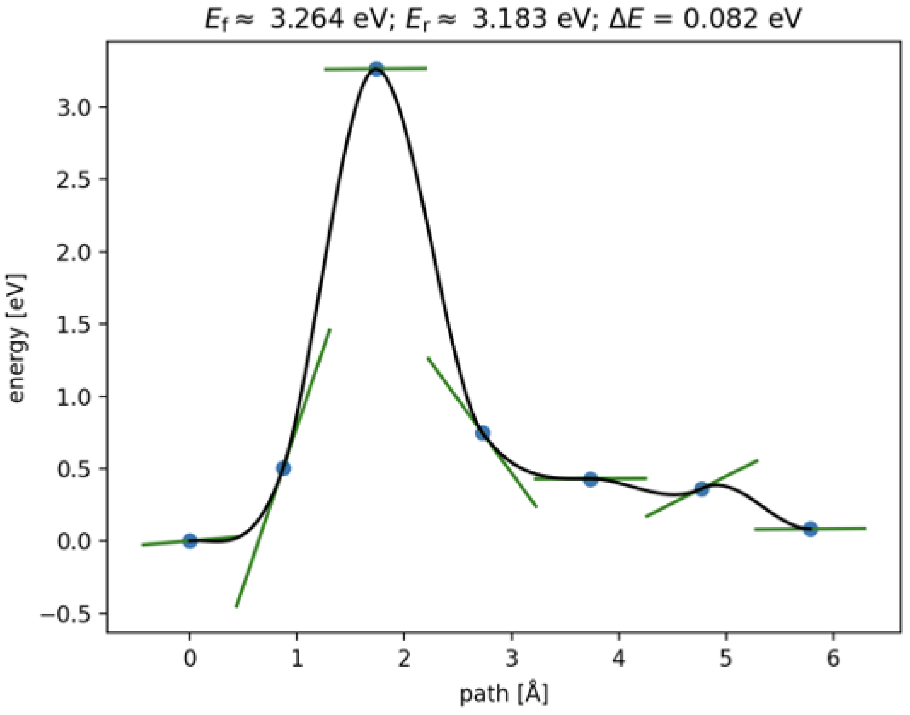

Figure S2. ML-NEB pathway for Sn(II) to Sn(IV) oxidation via H_2 abstraction as calculated with periodic DFT. A: Convergence criteria set to fmax =0.01 eV/Å uncertainty =0.05 eV. B: Convergence criteria set to fmax =0.05 eV/Å uncertainty =0.05 eV.

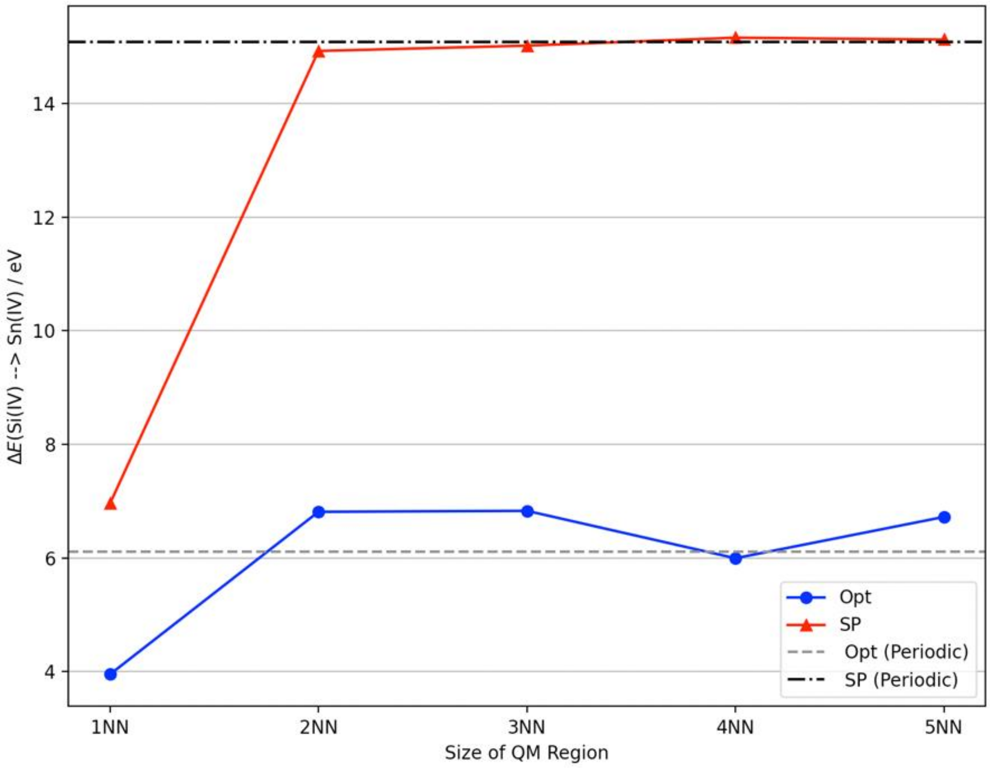

Figure S3. Plot of increasing size for QM region, from first to fifth nearest neighbour vs energy for single point calculations (SP) and geometry optimisation (Opt) of the oxidation of Sn(II) to Sn(IV). Energies of equivalent periodic calculations are also given as guide to convergence.

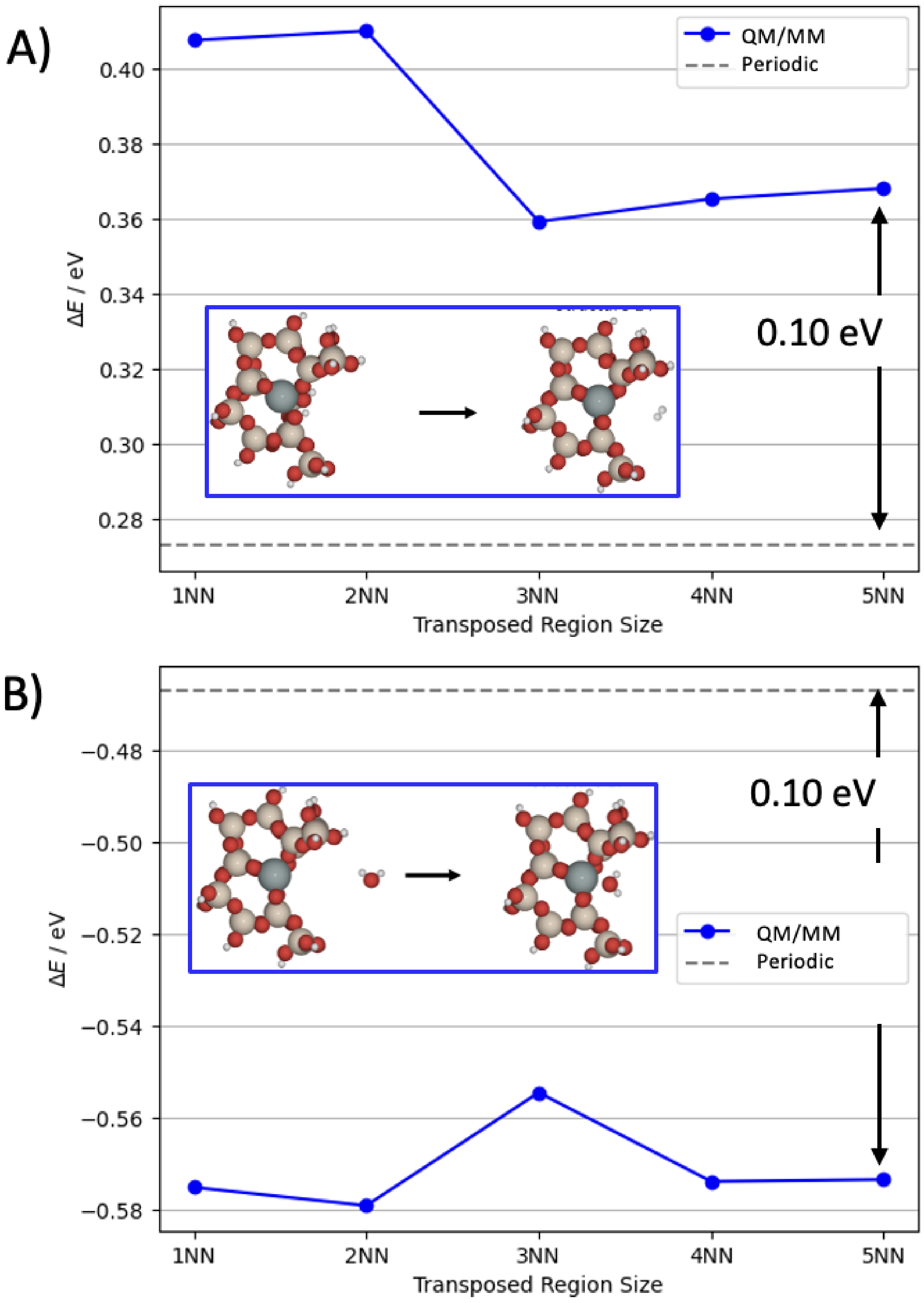

Figure S4: Plot of increasing size for transposed region from periodic models, from first to fifth nearest neighbour vs energy of geometry optimisation for A: oxidation of Sn(II) to Sn(IV). B: Adsorption of H_2O on Sn(IV). Energies of equivalent periodic calculations are also given as guide to convergence. Insets: visual representation of the structures for the processes.

Table S2: BSSE calculated for bidentate Sn(II) acetate in deAl-BEA for PBEsol+TS, PBE0+TS, and MP2.

Method	PBEsol+TS	PBE0+TS	MP2
**BSSE / eV**	-0.03	-0.04	-0.06

Table S3. Comparison of Sn-O_x distances of Sn(II) acetate optimised with PBEsol+TS and experiment. All distances are reported in Angstrom (Å).

Geometry

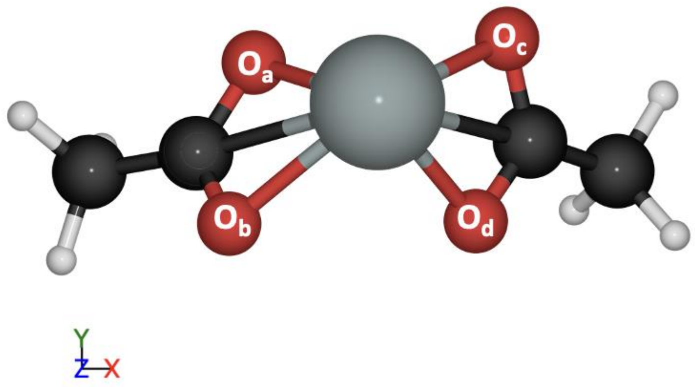

	**PBEsol+TS**	**Experiment[2]**
Sn-O_a	2.21	2.19
Sn-O_b	2.33	2.34
Sn-O_c	2.21	2.19
Sn-O_d	2.33	2.34

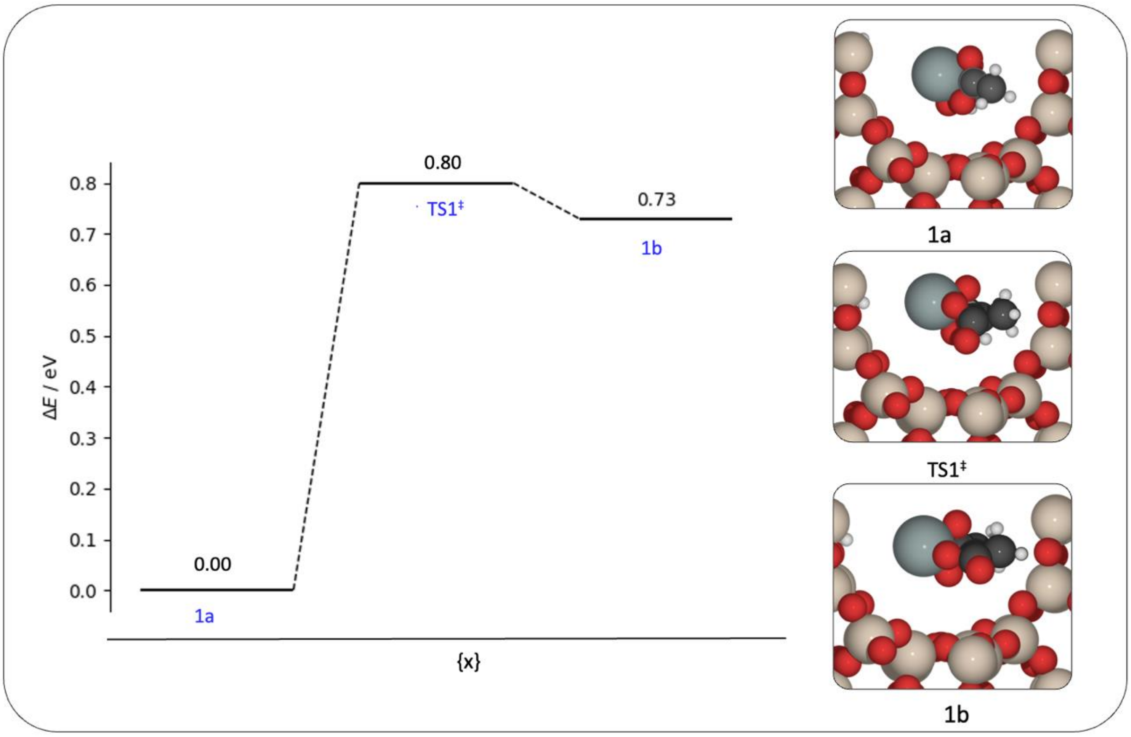

Figure S5. Graph of the reaction energies (ΔE) and kinetic barriers (E_{act}) for conversion between bidentate (1) and monodentate (1b) Sn(II) acetate within the BEA pore, as calculated with periodic DFT. Insets: visual representation of the structural intermediates, with structure numbers as labelled. The red, beige, white, black, and grey atoms represent O, Si, H, C, and Sn, respectively.

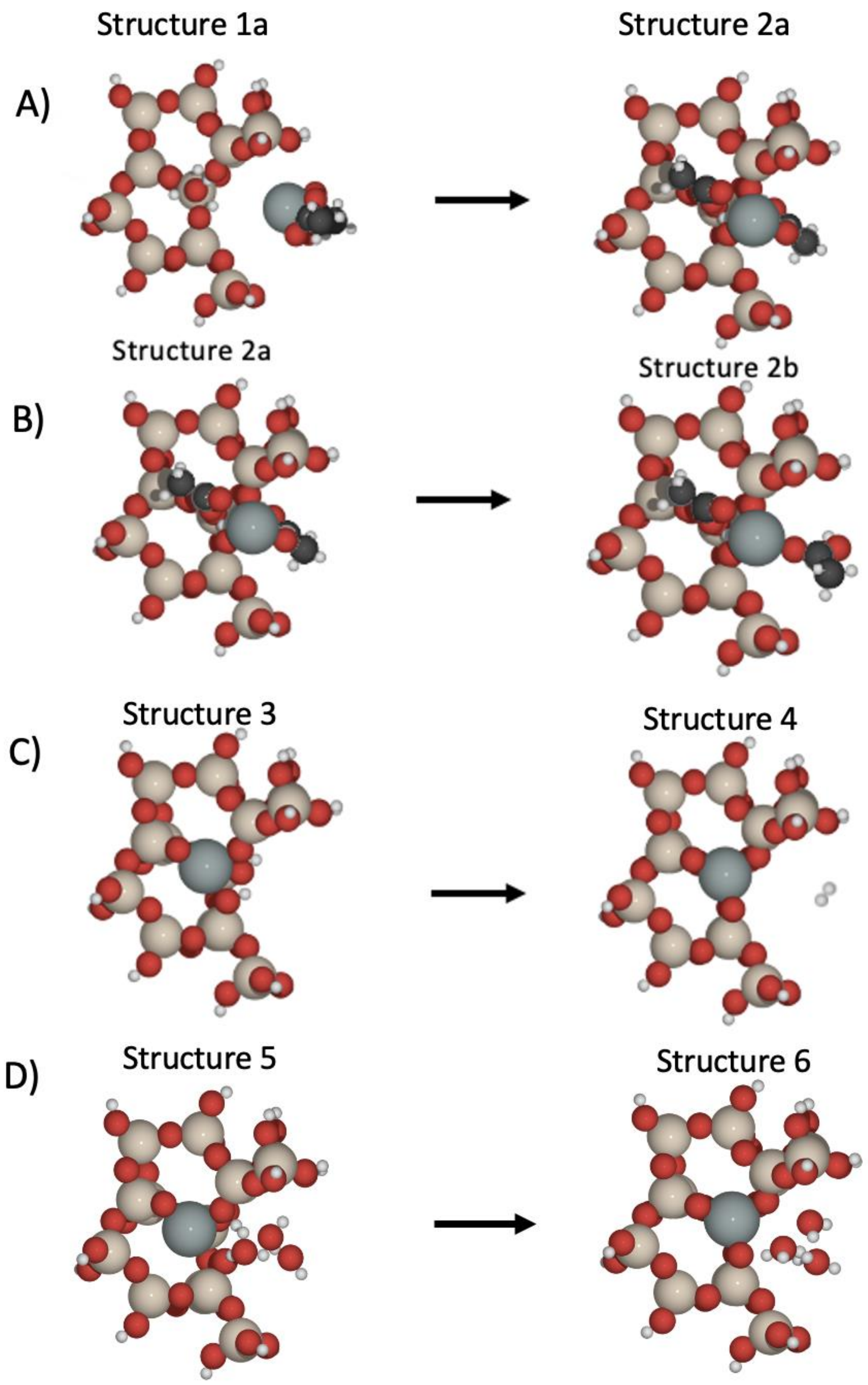
Structure 1a
Structure 2a
A)
Structure 2a
Structure 2b
B)
Structure 3
Structure 4
C)
Structure 5
Structure 6
D)

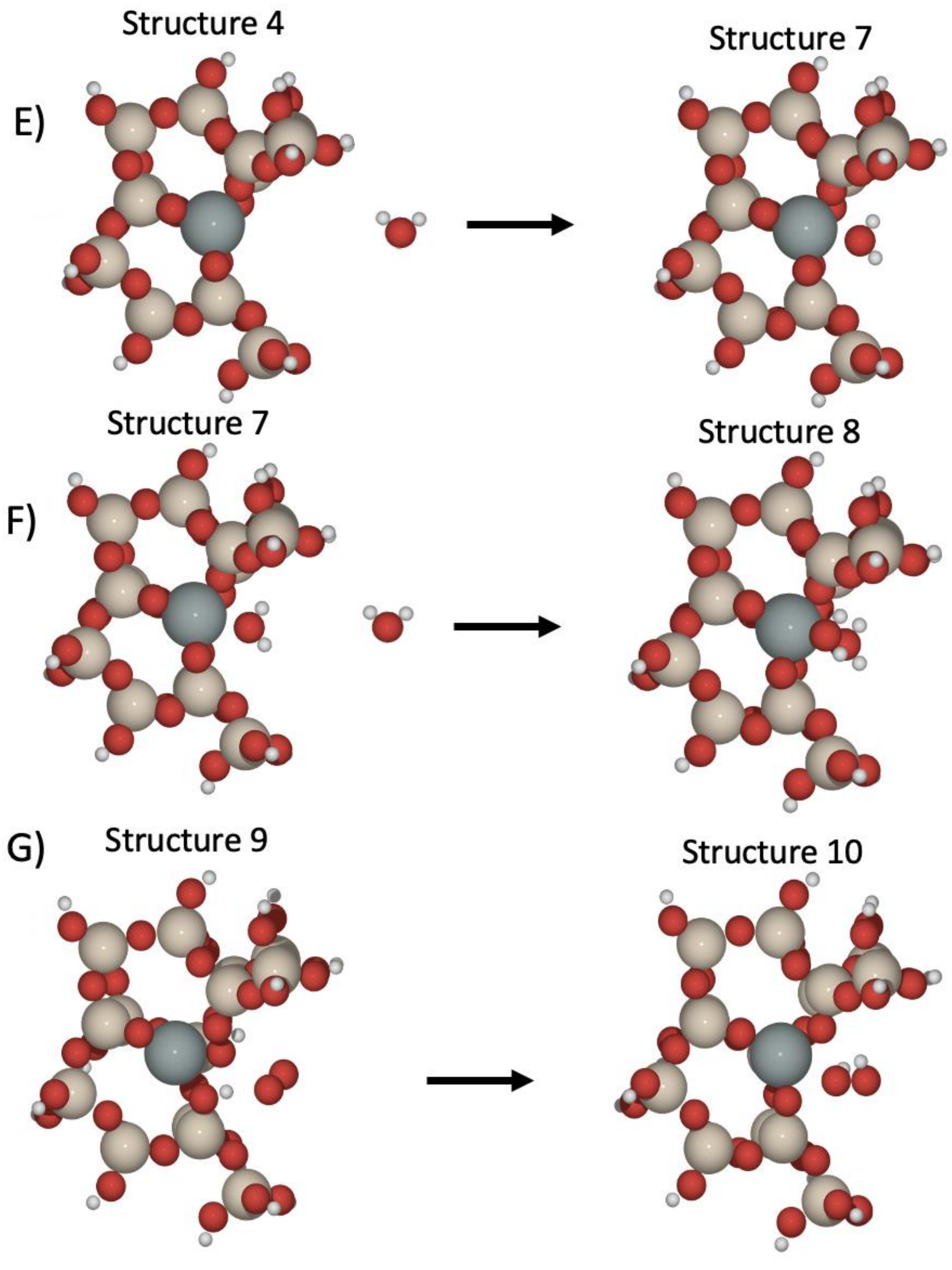

Figure S6. QM regions of calculations conducted with QM/MM methodology for stages in SSI and Sn-BEA synthesis; A: 1a → 2a B: 2a→ 2b. C: 3 → 4. D: 5→ 6. E: 4→ 7. F 7:→ 8. G: 9→ 10. The red, beige, white, black, and grey atoms represent O, Si, H, C, and Sn, respectively.

Table S4. Δ*E* (in eV) of processes as displayed in Figure S6 calculated with QM/MM.

**Process**	**QM Method**		
	**PBEsol + TS**	**PBE0 + TS**	**MP2**
**1a→ 2a**	-1.47	-1.59	-3.21
**2a→ 2b**	0.46	0.12	-0.04
**3→ 4**	0.39	0.21	-0.57
**5→ 6**	0.91	0.41	0.01
**4→ 7**	-0.21	-0.18	-0.73
**7→ 8**	1.35	1.53	0.83

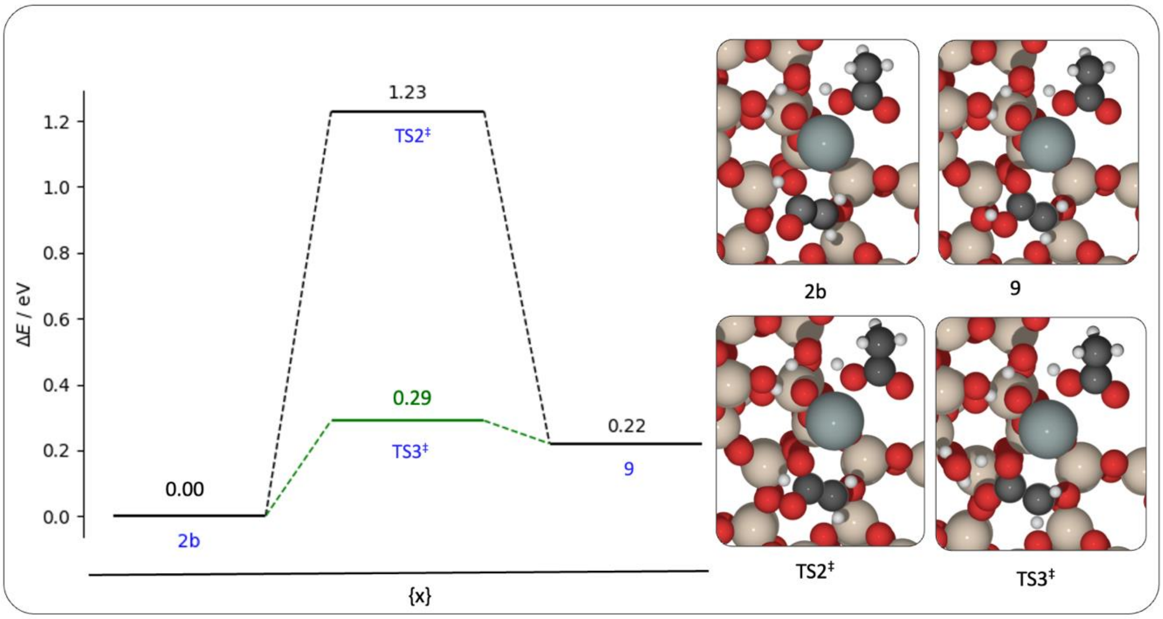

Figure S7. Graph of the reaction energies (Δ*E*) and kinetic barriers (E_{act}) for conversion between 2b and 9, as calculated with periodic DFT. Where the green line indicates pathway mediated by water. Insets: visual representation of the structural intermediates, with structure numbers as labelled. The red, beige, white, black, and grey atoms represent O, Si, H, C, and Sn, respectively.

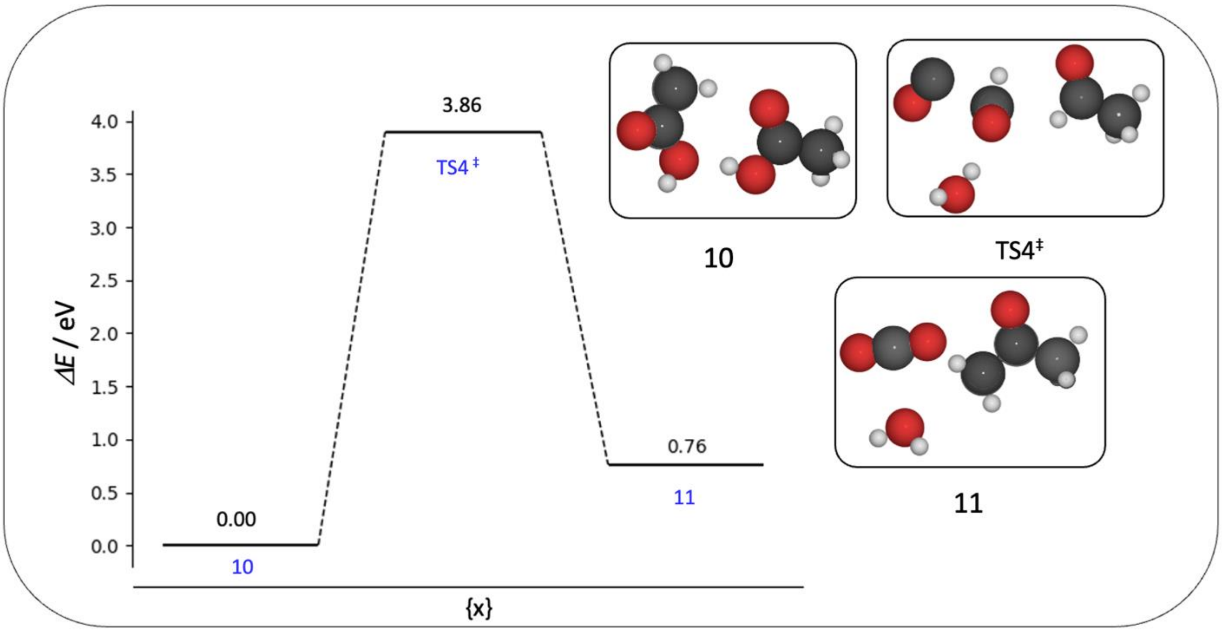

Figure S8. Graph of the reaction energies (Δ*E*) and kinetic barriers (E_{act}) for ketonisation of acetic acid to acetone and CO_2 and H_2O in the gas phase, as calculated with DFT (PBEsol+TS). Insets: visual representation of the structural intermediates, with structure numbers as labelled. The red, beige, white, black, and grey atoms represent O, Si, H, C, and Sn, respectively.

Table S5: Electron population and Mulliken charge for structures along the pathways for Sn oxidations via H_2 abstraction: without H_2O (3→ 4) and with three H_2O molecules (5→ 6), and with O_2, Sn(II) + O_2 (9) → Sn(IV) + H_2O_2 (10), as calculated with QM/MM (MP2).

Structure	Electron Population	Mulliken Charge
3	48.60	1.40
4	47.28	2.72
5	48.58	1.41
6	47.12	2.87
9	48.60	1.40
10	47.25	2.75

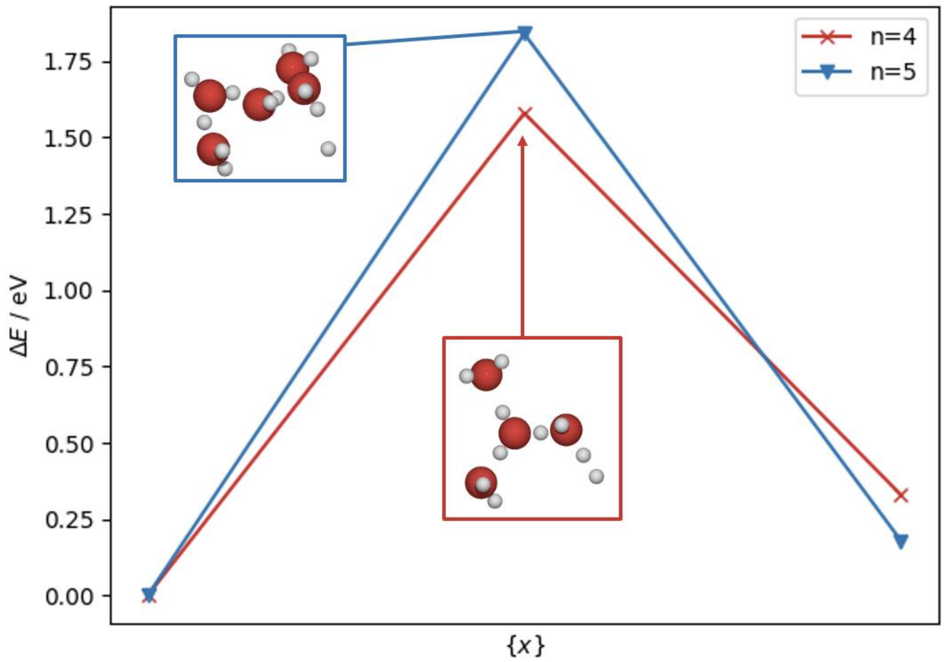

Figure S9. Graph of the reaction energies (ΔE) and kinetic barrier (E_{act}), as calculated with periodic-DFT, for oxidation of Sn(II) to Sn(IV) depending on the number (n) of H_2O molecules. Insets: transition states for the conversion of Sn(II) to Sn(IV), with production H_2 mediated by H_2O. Red and white atoms represent O and H, respectively.

References

(1) J. M. Newsam; M. M. J. Treacy; W. T. Koetsier; C. B. De Gruyter. Structural Characterization of Zeolite Beta. *Proc. R. Soc. Lond. A* **1988**, *420* (1859), 375–405.

(2) Smart, B. A.; Griffiths, L. E.; Pulham, C. R.; Robertson, H. E.; Mitzel, N. W.; Rankin, D. W. H. Molecular Structure of Tin(II) Acetate as Determined in the Gas Phase by Electron Diffraction and Ab Initio Calculations. *J. Chem. Soc., Dalton Trans.* **1997**, No. 9, 1565–1570.
